# Decoration of A-Ring of a Lupane-Type Triterpenoid with Different Oxygen and Nitrogen Heterocycles

**DOI:** 10.3390/molecules27154904

**Published:** 2022-07-31

**Authors:** Joana L. C. Sousa, Hélio M. T. Albuquerque, Armando J. D. Silvestre, Artur M. S. Silva

**Affiliations:** 1 LAQV-REQUIMTE, Department of Chemistry, University of Aveiro, 3810-193 Aveiro, Portugal; helio.albuquerque@ua.pt; 2 CICECO—Aveiro Institute of Materials, Department of Chemistry, University of Aveiro, 3810-193 Aveiro, Portugal; armsil@ua.pt

**Keywords:** triterpenoids, betulinic acid, oxygen heterocycles, nitrogen heterocycles, diastereoselectivity, Michael addition

## Abstract

Betulinic acid (BA) was used as starting building block to create a library of novel BA-derived compounds containing *O*- and *N*-heterocycles. Firstly, BA was converted into methyl betulonate (BoOMe), which was used as intermediate in the developed methodologies. 1,2-Oxazine-fused BoOMe compounds were obtained in 12–25% global yields through a Michael addition of nitromethane to methyl (*E*)-2-benzylidenebetulonate derivatives, followed by nitro group reduction and intramolecular cyclization. Remarkably, the triterpene acts as a diastereoselective inducer in the conjugate addition of nitromethane, originating only one diastereomer out of four possible ones. Furthermore, other oxygen and nitrogen-containing heterocycles were installed at the A-ring of BoOMe, affording 2-amino-3-cyano-4*H*-pyran-fused BoOMe, diarylpyridine-fused BoOMe and 1,2,3-triazole–BoOMe compounds, using simple and straightforward synthetic methodologies. Finally, BA was revealed to be a versatile starting material, allowing the creation of a molecular diversification of compounds containing a triterpenic scaffold and *O*- and *N*-heterocycles.

## 1. Introduction

**Figure 1:**
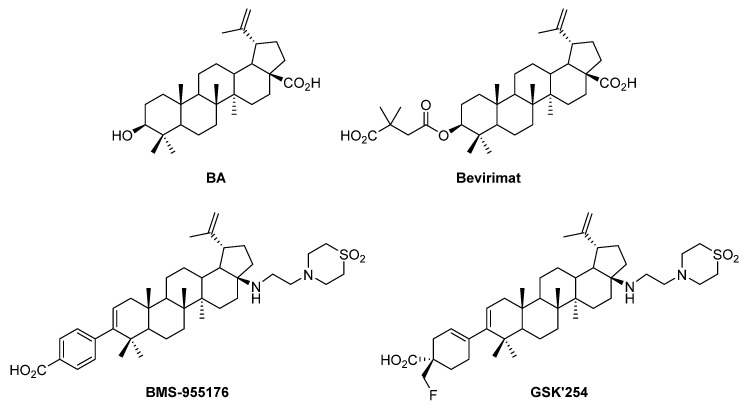
Structures of BA and three BA-based synthetic derivatives: bevirimat, BMS-955176 and GSK’254, known as potent anti-HIV agents.

Triterpenoids are plants’ secondary metabolites with a variety of promising biological activities. In particular, betulinic acid (BA), which is a lupane-type pentacyclic triterpenic acid (Figure 1), is commonly isolated from birch (*Betula* spp.) bark, displaying important biological activities [1,2]. Among triterpenic acids, BA gained special attention from the scientific community from the moment that two of its synthetic derivatives, namely bevirimat (Myriad Genetics) and BMS-955176 (GSK) (Figure 1), were known as efficient anti-HIV drugs, reaching phase IIb clinical trials [3,4]. Meanwhile, GSK pursued its study in the development of HIV maturation inhibitors and produced another interesting molecule with a similar structure to BMS-955176 known as GSK’254 (Figure 1), which is a phase IIb clinical candidate [5].

BA proved to be an attractive scaffold for chemical upgrading [6], including its functionalization with oxygen- and nitrogen-containing heterocycles with very encouraging biological properties, especially as antitumor agents [6,7,8,9].

**Figure 2:**
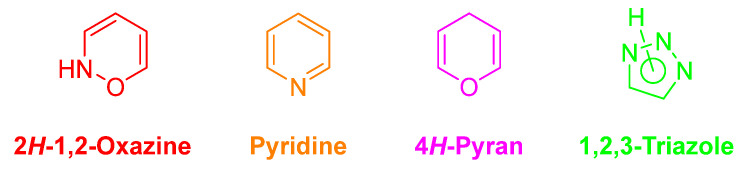
2*H*-1,2-Oxazine, pyridine, 4*H*-pyran and 1,2,3-triazole cores.

Heterocyclic compounds are considered as the backbone of drug discovery efforts. The ability to access heterocyclic frameworks in a general and rapid manner is essential for medicinal chemists, which would allow them to drive structure−activity relationships (SARs) and to address physicochemical and/or disposition liabilities of common heterocyclic templates. In this context, we selected four types of heterocycles, namely 2*H*-1,2-oxazines, 4*H*-pyrans, pyridines and 1,2,3-triazoles (Figure 2), due to their widely recognized impact in medicinal chemistry projects either as pharmacophores and/or modulators of physicochemical properties.

Oxazines and their derivatives are heterocyclic compounds containing one oxygen atom and one nitrogen atom (Figure 2) [10]. These heterocycles have special interest because they constitute an important class of non-natural and natural products, which display useful biological properties [11,12,13] as well as interesting photophysical and lasing properties [14,15]. To the best of our knowledge, this six-membered *N,O*-heterocycle was never installed before in the BA skeleton. Therefore, a reliable synthetic strategy for its installation in the A-ring of the BA skeleton is quite desirable and will open numerous possibilities for the synthesis of novel families of fused 1,2-oxazine–BA compounds.

Among the plethora of *O*-heterocycles, the 4*H*-pyran scaffold (Figure 2) stands out as it can be found in coumarins, benzopyrans, sugars, flavonoids and xanthones, among others [16]. It also can be regarded as a key pharmacophore playing a pivotal role in several biological activities, especially as anticancer agents [16]. Within the family of 4*H*-pyrans, special attention should be given to the 2-amino-3-cyano-4*H*-pyran derivatives, as part of 2-amino-3-cyano-4*H*-chromenes, which is a key scaffold in the anticancer agent crolibulin (EPC2407) currently in phase I/II clinical trials [17]. Once again, to the best of our knowledge, it is the first time that this specific type of 4*H*-pyran was fused to a lupane-type pentacyclic triterpenoid.

**Scheme 1:**
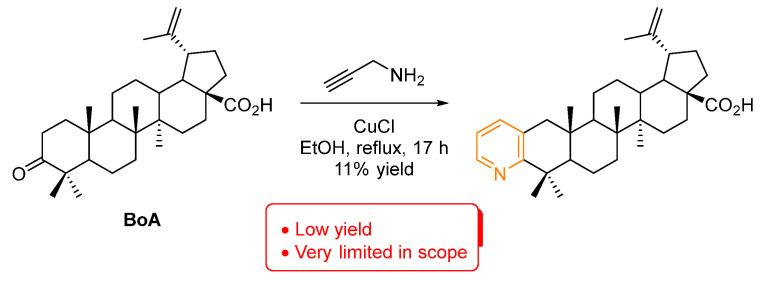
Synthesis of a pyridine-fused BA compound [20].

Concerning pyridines (Figure 2), they are one of the most prevalent examples of biologically active heterocycles, being the second most common ring in small molecule drugs. This is quite understandable since the pyridine ring is often used as an isosteric and/or bioisosteric replacer of phenyl rings [18,19]. There is only one example of a pyridine-fused BA compound [20]. It was prepared by treatment of betulonic acid (BoA) with propargyl amine via an intermediate enamine, which in the presence of CuCl catalyst performs a ring closure to form pyridine (Scheme 1). However, this method is quite limited both in reaction yield and the ability to install different substitution patterns. Nevertheless, this pyridine-fused BA compound was further tested against the axenic amastigotes of *Leishmania donovani* and inhibited 88% at 50 µM, but only 26% at 15 µM, demonstrating the potential of the pyridine installation in the BA scaffold.

Bioisosteres can be categorized into classical and nonclassical subtypes, with pyridines being a classical subtype. In this field, we also should look upon the tremendous role of 1,2,3-triazoles (Figure 2), as nonclassical surrogates of amide bond-containing molecules [21]. Despite the 1,2,3-triazole moiety being present in several drugs, they were first developed as antifungal agents such as fluconazole, isavuconazole and itraconazole, followed by the widening of their application in the treatment of various medicinal conditions [21]. Among chosen heterocycles, 1,2,3-triazole-containing lupane-type pentacyclic triterpenoids are the most found ones in the literature [6,9]. The employed methodology to prepare 1,2,3-trizole-linked BA compounds is the copper-catalyzed azide-alkyne cycloaddition (CuAAC), which allowed the derivatization of BA at positions 2, 3, 28 and 30 and the synthesis of BA-based compounds with anti-inflammatory, anticancer and anti-hepatitis C virus properties, among others [6].

**Scheme 2:**
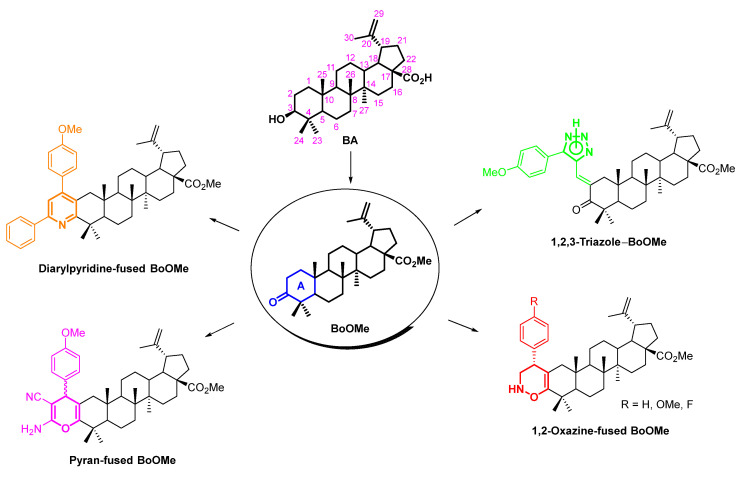
Structures of target oxygen/nitrogen-heterocycle-containing BoOMe compounds, obtained from natural BA.

Herein, aiming at valorizing biomass derived compounds, our goal was to decorate BA with 2*H*-1,2-oxazine, 4*H*-pyran, pyridine and 1,2,3-triazole moieties at its A-ring in order to obtain oxygen and nitrogen heterocycle-containing methyl betulonate (BoOMe) compounds (Scheme 2). To do so, simple and straightforward synthetic methodologies were established using easily accessible and cheap reagents.

## 2. Results and Discussion

### 2.1. Diastereoselective Michael Addition of Nitromethane to Methyl (E)-2-Benzylidenebetulonate Derivatives towards the Synthesis of 1,2-Oxazine-Fused BoOMe Compounds

**Scheme 3:**
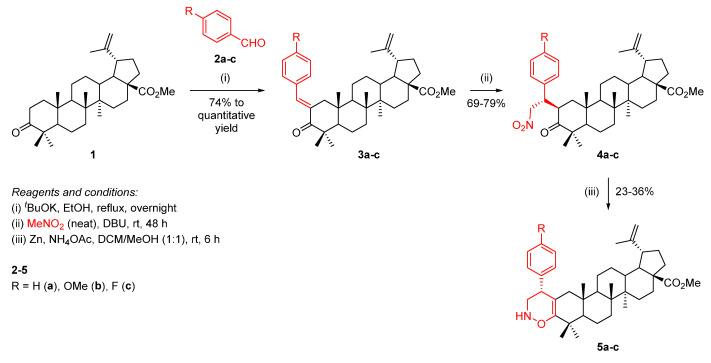
Synthesis of 1,2-oxazine-fused BoOMe compounds **5a-c**.

In order to fuse a 1,2-oxazine unit to the A-ring of BoOMe, a three-step approach was developed (Scheme 3). Starting from a base-promoted aldol condensation reaction of BoOMe (**1**) with benzaldehydes **2a-c**, methyl (*E*)-2-benzylidenebetulonate derivatives **3a-c** were obtained, which underwent a Michael addition of nitromethane, affording Michael adducts **4a-c**. Then, the subsequent reduction in their nitro group and nucleophilic intramolecular ring cyclization led to the formation of the desired 1,2-oxazine-fused BoOMe compounds **5a-c**.

Firstly, BoOMe (**1**) was synthesized from BA through the esterification of its 17-COOH group and oxidation of its 3-OH group (Scheme 2) [22]. Then, the required methyl (*E*)-2-benzylidenebetulonate derivatives **3a-c** were obtained in good (74% for **3c**) to quantitative (for **3a,b**) yields through the aldol condensation reaction of BoOMe (**1**) with benzaldehydes **2a-c** in the presence of potassium *t*-butoxide (*^t^*BuOK) as a base, in refluxing ethanol, overnight (Scheme 3).

**Figure 3:**
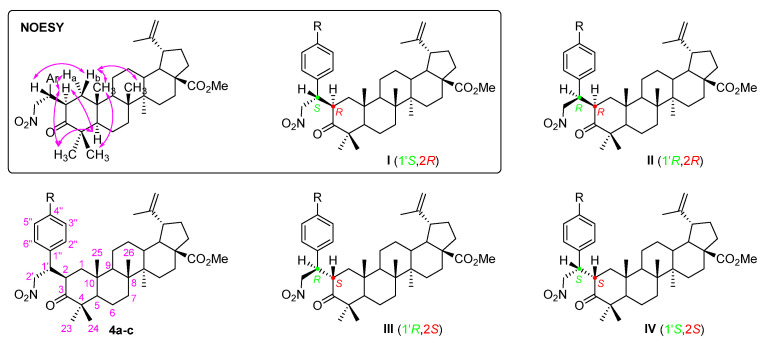
Adopted numbering system, structures of four possible diastereomers **I** to **IV** and main NOE effects (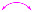) for Michael adducts **4a-c**. Ar = aryl group.

Secondly, a Michael addition of nitromethane to α,β-unsaturated carbonyl compounds **3a-c** was accomplished in the presence of 1,8-diazabicyclo[5.4.0]undec-7-ene (DBU) as a base, under neat conditions (i.e., nitromethane was used as reagent as well as solvent), affording Michael adducts **4a-c** in good yields (69–79%). This reaction led to the creation of two new stereocenters and, consequently, it would be expected that a mixture of diastereomers **I** to **IV** would be obtained (Figure 3). However, a single diastereomer was formed. Usually, the asymmetric Michael addition of nitromethane involves the use of complex organocatalysts [23,24,25], however, in our particular case, their use was not required, since the observed diastereoselectivity was inducted by the defined stereochemistry of the triterpene scaffold.

The relative stereochemistry of the obtained diastereomer was established by NOESY experiments (Figure S7 for derivative **4b**). There was evidence of close proximity of: H-2 with H_a_-1, H-5 and H-23, and H-1′ with H_b_-1 (Figure 3 inset), which suggests that diastereomer **I** was the most plausible obtained structure.

Lastly, the desired 1,2-oxazine-fused BoOMe compounds **5a-c** were obtained in poor yields (23–36%) through the reaction of Michael adducts **4a-c** with an excess of zinc and ammonium acetate (NH_4_OAc) in a mixture of dichloromethane (DCM)/methanol (1:1) at room temperature for 6 h (Scheme 3). The low yields can be justified by the formation of an identified by-product together with the desired product.

**Scheme 4:**
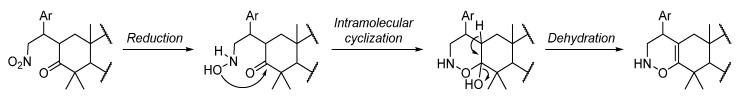
Proposed tandem reaction pathway for the formation of 1,2-oxazine-fused BoOMe compounds **5a-c**. Ar = aryl group.

Previous studies developed by our group disclosed that the incomplete reduction of the nitro group led to an intramolecular aza-nucleophilic addition, originating Δ^1^-pyrroline-*N*-oxide or *N*-hydroxypyrrolidine derivatives depending on the substrate [23,26]. Interestingly, herein, it is worth mentioning that the reaction cascade involves the reduction of the nitro group to a hydroxylamino group, followed by an intramolecular oxa-nucleophilic addition and dehydration, leading to the formation of a 1,2-oxazine-type ring as depicted in Scheme 4.

**Figure 4:**
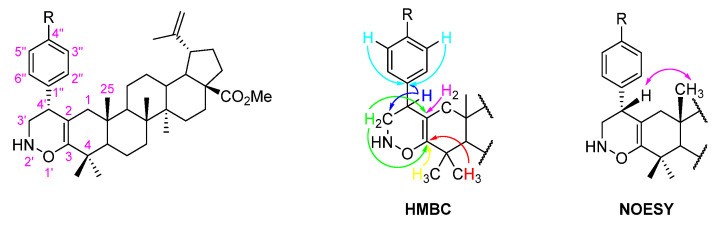
Adopted numbering system and main HMBC connectivities and NOE effects (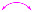) observed for 1,2-oxazine-fused BoOMe compounds **5a-c**.

The formation of 1,2-oxazine-fused BoOMe compounds **5a-c** was confirmed by 1D and 2D NMR techniques. Thus, in their ^13^C NMR spectra, the appearance of two non-protonated carbons at δ_C_ 92.6–98.9 and 180.8–182.1 ppm, which were unequivocally assigned as C-2 and C-3, respectively, by their HMBC connectivities with H-1 and H-3′, and H-3′ and 4-(C*H*_3_)_2_ (Figure 4), proves the fusion of a 1,2-oxazine-type ring to the A-ring of BoOMe. In addition, the band at 3300–3350 cm^−1^ observed in the ATR-FTIR spectrum of compound **5b** confirms the presence of a secondary amino group in the structure of this compound (Figure S14). Furthermore, the relative configuration of the C-4′ stereocenter was unveiled through NOESY spectroscopy (Figure 4). Close proximity was observed between H-4′ and H-25 in the NOESY spectra of compounds **5a-c** (Figure S17 illustrates the main NOE effects for derivative **5c**).

### 2.2. Synthesis of Other Oxygen and Nitrogen Heterocycles-Containing BoOMe Compounds Using Different Synthetic Methodologies

#### 2.2.1. Michael Addition of Malononitrile to Methyl (*E*)-2-(4-Methoxybenzylidene)betulonate towards the Synthesis of a Pyran-Fused BoOMe Compound

**Scheme 5:**
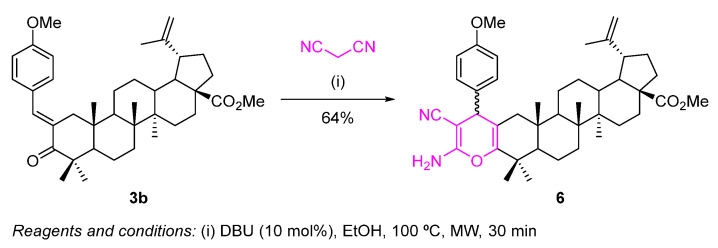
Synthesis of a highly functionalized 4*H*-pyran-fused BoOMe compound **6**.

Using the methyl (*E*)-2-(4-methoxybenzylidene)betulonate (**3b**) as substrate, we were able to fuse a highly functionalized 4*H*-pyran to the A-ring of the triterpene scaffold through a one-pot microwave (MW)-assisted Michael addition of malononitrile to the α,β-unsaturated carbonyl derivative **3b**, followed by an intramolecular cyclization (Scheme 5).

This reaction was performed under classical reaction conditions described in the literature to prepare 2-amino-3-cyano-4*H*-pyrans [27]. Thus, using an excess of malononitrile, in the presence of a catalytic amount of DBU, at 100 °C under MW irradiation for 30 min, the desired 4*H*-pyran-fused BoOMe compound **6** was obtained in 64% yield (Scheme 5), although there were still traces of starting material **3b** in the crude reaction media.

**Figure 5:**
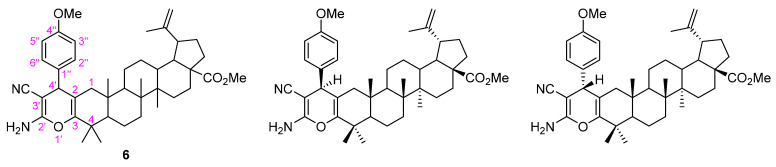
Adopted numbering system and structures of two diastereomers of the pyran-fused BoOMe compound **6**.

Contrarily to what was previously observed for the Michael addition of nitromethane, herein, the conjugate addition of malononitrile to the α,β-unsaturated carbonyl derivative **3b** did not occur in a diastereoselective manner, since compound **6** was obtained as an inseparable mixture of two diastereomers (Figure 5).

This could be demonstrated by the two sets of signals assigned to the *p*-substituted protons observed in the aromatic region of the ^1^H NMR spectrum (Figure S18). In addition, other important features should be highlighted in the ^1^H NMR spectrum of **6**, namely the resonances of protons from the 2-amino-3-cyano-4*H*-pyran moiety, as follows: (i) a broad singlet at δ_H_ 4.35 ppm corresponding to the 2′-N*H*_2_ group, and (ii) two singlets at δ_H_ 3.64 and 3.63 ppm corresponding to H-4′ of both diastereomers. In the ^13^C NMR spectrum of **6** (Figure S19), it can be observed that several pairs of resonances can be attributed to the same carbon in different diastereomers, notably: δ_C_ 120.3 and 120.4 ppm assigned to 3′-*C*≡N group; δ_C_ 60.88 and 60.92 ppm to C-3′; δ_C_ 147.82 and 147.91 ppm to C-3; δ_C_ 106.4 and 107.1 ppm to C-2; and lastly, one resonance at δ_C_ 159.0 ppm assigned to C-2′.

#### 2.2.2. Kröhnke Pyridine Synthesis—Synthesis of a Diarylpyridine-Fused BoOMe Compound

**Scheme 6:**
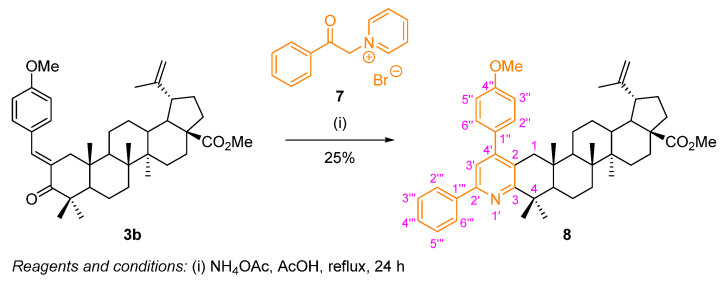
Synthesis of the diarylpyridine-fused BoOMe compound **8**.

A diarylpyridine-fused BoOMe compound **8** was prepared following a Kröhnke synthetic route (Scheme 6). Once again, using the methyl (*E*)-2-(4-methoxybenzylidene)betulonate (**3b**) as starting material, the reaction was performed in the presence of an excess of the Kröhnke salt **7** and ammonium acetate, in refluxing acetic acid for 24 h. Compound **8** was obtained in poor yield (25%), still recovering some starting material **3b**. In the literature, for simpler triarylpyridine derivatives, prepared through a similar methodology, it was observed that the presence of strong electron-donating groups, such as dimethylamino or methoxy groups, did not favor the reaction, since the corresponding triarylpyridines were obtained in poor yields (24–37%) [28]. Therefore, this might have happened for the diarylpyridine-fused BoOMe compound **8**, as the starting material **3b** also possesses a strong electron-donating methoxy substituent.

The key structural features found in the ^1^H NMR spectrum of **8** (Figure S20) comprise the singlet at δ_H_ 7.39 ppm corresponding to the resonance of H-3′ of the pyridine moiety, and additionally, further multiplets at the aromatic region are observed, corresponding to the resonances of the protons from the phenyl ring (resulting from Kröhnke salt).

#### 2.2.3. 1,3-Dipolar Cycloaddition of Sodium Azide to a Methyl (*E*)-2-(3-Phenylprop-2-yn-1-ylidene)betulonate Derivative—Synthesis of a 1,2,3-Triazole–BoOMe Compound

**Scheme 7:**
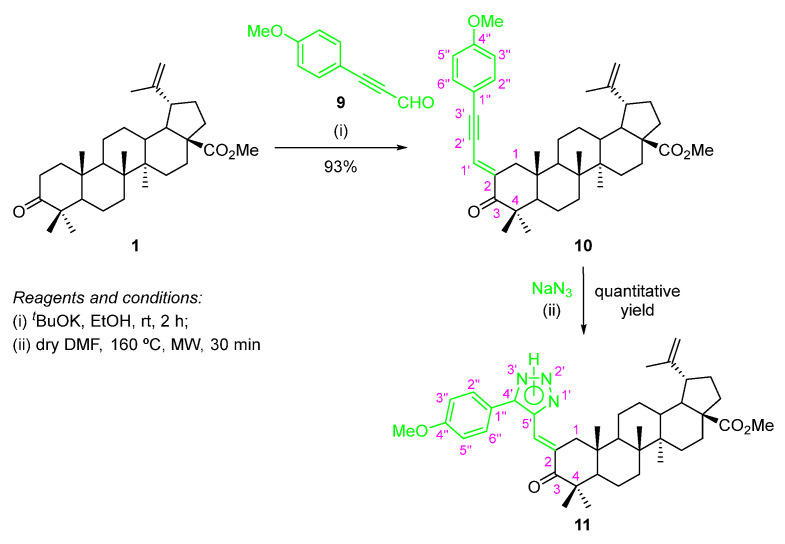
Synthesis of methyl (*E*)-2-[(5-phenyl-2*H*-1,2,3-triazol-4-yl)methylene]betulonate compound **11**.

A BoOMe-based compound containing a 1,2,3-triazole ring was synthesized through a two-step approach (Scheme 7).

The first step involved a base-promoted aldol condensation reaction of BoOMe (**1**) with 3-(4-methoxyphenyl)propiolaldehyde (**9**) using *^t^*BuOK as a base (Scheme 7), affording the methyl (*E*)-2-[3-(4-methoxyphenyl)prop-2-yn-1-ylidene]betulonate (**10**) in excellent yield (93%). The required 3-(4-methoxyphenyl)propiolaldehyde (**9**), which are not commercially available, had to be synthesized according to a two-step procedure described in the literature [29]. The main feature in the ^1^H NMR spectrum of compound **10** is the resonance corresponding to the methine proton (=C*H*–), which appears as a broad doublet at δ_H_ 6.75 ppm. Additionally, in its ^13^C NMR spectra, the resonances of C-2′ and C-3′ of the triple bond (C2′≡C3′) can be observed at δ_C_ 86.2 and 103.2 ppm, respectively.

The following step involved the 1,3-cycloaddition reaction of intermediate **10** with sodium azide under MW irradiation, giving rise to the desired methyl (*E*)-2-[(5-phenyl-2*H*-1,2,3-triazol-4-yl)methylene]betulonate **11** in quantitative yield (Scheme 7). In the ^13^C NMR spectrum of this compound, the most important feature is the C-4′ and C-5′ resonances from the 1,2,3-triazole moiety, at δ_C_ 140.0 and 146.4 ppm, respectively.

## 3. Materials and Methods

### 3.1. General Remarks

The melting points were measured with a Büchi Melting Point B-540 apparatus and are uncorrected. NMR spectra were recorded with Bruker Avance 300 (300.13 MHz for ^1^H and 75.47 MHz for ^13^C) or Bruker Avance 500 (500.13 MHz for ^1^H and 125.77 MHz for ^13^C) spectrometers, in CDCl_3_ as solvent. Chemical shifts (δ) are reported in ppm and coupling constants (*J*) in Hz; the internal standard was tetramethylsilane (TMS). Unequivocal ^13^C assignments were made with the aid of 2D *g*HSQC and *g*HMBC (delays for one-bond and long-range *J*_C/H_ couplings were optimized for 145 and 7 Hz, respectively) experiments. Positive-ion ESI mass spectra were acquired with a Q-TOF 2 or an LTQ Orbitrap XL spectrometer. MW-assisted reactions were carried out in a CEM Discover SP apparatus. Preparative TLC was performed with Macherey-Nagel silica gel G/UV254. All chemicals and solvents were obtained from commercial sources and used as received or dried by standard procedures. BA was purchased from Aktin Chemicals, Inc. (Chengdu, China). BoOMe (**1**) was prepared from BA following a methodology described in the literature [22].

### 3.2. Synthesis of Methyl (E)-2-Benzylidenebetulonate Derivatives ***3a-c*** and Methyl (E)-2-[3-(4-Methoxyphenyl)prop-2-yn-1-ylidene]betulonate *(**10**)*

*^t^*BuOK (71.7 mg, 0.639 mmol) was added to a solution of BoOMe (**1**) (100 mg, 0.213 mmol) in EtOH (5 mL). After 15 min, the appropriate benzaldehyde **2a-c** or 3-(4-methoxyphenyl)propiolaldehyde (**9**) (0.256 mmol) was added, and the reaction mixture was stirred at reflux overnight for **3a-c** or at room temperature for 2 h for **10**. After this period, it was poured onto ice (10 g) and H_2_O (5 mL), and the pH was adjusted to 1 with diluted HCl. Then, this aqueous solution was extracted twice with DCM and the organic layer was dried over anhydrous Na_2_SO_4_. The solvent was evaporated to dryness and the obtained residue was purified by preparative TLC using a mixture of Hex/EtOAc (10:1) as eluent. Methyl (*E*)-2-benzylidenebetulonate (**3a**) and methyl (*E*)-2-(4-methoxybenzylidene)betulonate (**3b**) presented similar data to the one described in the literature [22].

*Methyl (E)-2-(4-fluorobenzylidene)betulonate (**3c**).* Colorless oil (90.6 mg, 74% yield). ^1^H NMR (300.13 MHz, CDCl_3_): δ = 0.79–2.32 (m, 21H), 0.79 (s, 3H, H-25), 0.96 and 1.02 (2 s, 6H, H-26,27), 1.12 and 1.15 (2 s, 6H, H-23,24), 1.72 (s, 3H, H-30), 2.97–3.06 (m, 2H), 3.68 (s, 3H, 28-OC*H*_3_), 4.65 (dd, *J* = 1.4 and 2.3 Hz, 1H, H-29), 4.77 (d, *J* = 2.3 Hz, 1H, H-29), 7.10 (t, *J* = 8.7 Hz, 2H, H-3′,5′), 7.40 (dd, *J* = 5.5 and 8.7 Hz, 2H, H-2′,6′), 7.44 (br s, 1H, −C*H*=) ppm. ^13^C NMR (75.47 MHz, CDCl_3_): δ = 14.6 and 15.4 (C-26,27), 15.8 (C-25), 19.5 (C-30), 20.4, 21.7, 22.4 and 29.5 (C-23,24), 25.6, 29.6, 30.7, 32.1, 33.1, 36.5, 36.9, 38.3, 40.5, 42.5, 44.4, 45.2, 46.9, 48.5, 49.4, 51.3 (28-O*C*H_3_), 52.8, 56.6, 109.5 (C-29), 115.6 (d, *J* = 21.5 Hz, C-3′,5′), 132.1 (d, *J* = 2.7 Hz, C-1′), 132.2 (d, *J* = 8.1 Hz, C-2′,6′), 133.9 (d, *J* = 1.9 Hz, C-2), 136.1 (−*C*H=), 150.7 (C-20), 162.5 (d, *J* = 249.8 Hz, C-4′), 176.6 (C-28), 208.1 (C-3) ppm. HRMS-ESI *m/z* calcd for [C_38_H_51_FO_3_ + H]^+^: 575.3900, found: 575.3891.

*Methyl (E)-2-[3-(4-methoxyphenyl)prop-2-yn-1-ylidene]betulonate *(**10**)*.* White solid (121 mg, 93% yield). Mp 197–198 °C. ^1^H NMR (300.13 MHz, CDCl_3_): δ = 0.76–2.33 (m, 21H), 0.83 (s, 3H, H-25), 0.97 and 1.01 (2 s, 6H, H-26,27), 1.10 (s, 6H, H-23,24), 1.72 (s, 3H, H-30), 2.98–3.16 (m, 2H), 3.68 (s, 3H, 28-OC*H*_3_), 3.84 (s, 3H, 4″-OC*H*_3_), 4.63–4.64 (m, 1H, H-29), 4.77 (d, *J* = 2.0 Hz, 1H, H-29), 6.75 (br d, *J* = 8.9 Hz, 1H, H-1′), 6.89 (d, *J* = 8.9 Hz, 2H, H-3″,5″), 7.44 (d, *J* = 8.9 Hz, 2H, H-2″,6″) ppm. ^13^C NMR (75.47 MHz, CDCl_3_): δ = 14.6 and 15.5 (C-26,27), 15.9 (C-25), 19.5 (C-30), 20.3, 21.6, 22.5 and 29.0 (C-23,24), 25.6, 29.7, 30.7, 32.1, 33.1, 36.5, 36.9, 38.3, 40.5, 42.5, 44.5, 45.2, 46.9, 48.3, 49.4, 51.3 (28-O*C*H_3_), 53.3, 55.4 (4″-O*C*H_3_), 56.6, 86.1 (C-2′), 103.2 (C-3′), 109.6 (C-29), 114.1 (C-3″,5″), 114.9 (C-1″), 118.8 (C-1′), 133.5 (C-2″,6″), 143.1 (C-2), 150.6 (C-20), 160.2 (C-4″), 176.6 (C-28), 206.1 (C-3) ppm. HRMS-ESI *m/z* calcd for [C_41_H_54_O_4_ + H]^+^: 611.4100, found: 611.4079.

### 3.3. Synthesis of Methyl 2-(2-Nitro-1-phenylethyl)betulonate Derivatives ***4a-c***

DBU (0.51 mmol, 76 µL) was added to a solution of the appropriate methyl (*E*)-2-benzylidenebetulonate derivative **3a-c** (0.17 mmol) in dry MeNO_2_ (0.8 mL). A few drops of DCM had to be added for the complete dissolution of starting material **3a**. The reaction mixture was stirred at room temperature for 48 h. After this period, the reaction crude was dissolved in DCM and then purified by preparative TLC using a mixture of Hex/EtOAc (6:1) as eluent.

*Methyl 2-(2-nitro-1-phenylethyl)betulonate *(**4a**)*.* Colorless oil (72 mg, 69% yield). ^1^H NMR (300.13 MHz, CDCl_3_): δ = 0.66 (s, 3H, H-25), 0.79–2.24 (m, 22H), 0.85 and 0.93 (2 s, 6H, H-26,27), 1.05 and 1.13 (2 s, 6H, H-23,24), 1.65 (s, 3H, H-30), 2.91–2.96 (m, 1H), 3.25 (q, *J* = 10.0 Hz, 1H, H-2), 3.60 (dt, *J* = 4.7 and 9.2 Hz, 1H, H-1′), 3.65 (s, 3H, 28-OC*H*_3_), 4.56–4.57 (m, 1H, H-29), 4.64–4.71 (m, 2H, H-2′ and H-29), 4.77 (dd, *J* = 4.3 and 12.3 Hz, 1H, H-2′), 7.12–7.36 (m, 5H, H-2″,3″,4″,5″,6″) ppm. ^13^C NMR (125.77 MHz, CDCl_3_): δ = 14.6 and 15.2 (C-26,27), 18.6 (C-25), 19.3 and 28.6 (C-23,24), 19.4 (C-30), 20.0, 21.8, 25.4, 29.6, 30.6, 32.0, 32.7, 36.9, 37.1, 38.3, 40.6, 42.4, 42.7 (C-2), 44.8 (C-1′), 46.9, 47.5, 48.3, 49.3, 50.0, 51.3 (28-O*C*H_3_), 52.4, 56.5, 79.0 (C-2′), 109.6 (C-29), 127.8 (C-4″), 128.1 (C-3″,5″), 129.0 (C-2″,6″), 137.7 (C-1″), 150.6 (C-20), 176.6 (C-28), 219.2 (C-3) ppm. HRMS-ESI *m/z* calcd for [C_39_H_55_NO_5_ + H]^+^: 618.4158, found: 618.4173.

*Methyl 2-[1-(4-methoxyphenyl)-2-nitroethyl]betulonate *(**4b**)*.* Colorless oil (87 mg, 79% yield). ^1^H NMR (500.13 MHz, CDCl_3_): δ = 0.65 (s, 3H, H-25), 0.79–2.25 (m, 22H), 0.86 and 0.93 (2 s, 6H, H-26,27), 1.05 and 1.12 (2 s, 6H, H-23,24), 1.67 (s, 3H, H-30), 2.92–2.98 (m, 1H), 3.20 (q, *J* = 10.1 Hz, 1H, H-2), 3.55 (dt, *J* = 4.1 and 9.2 Hz, 1H, H-1′), 3.65 (s, 3H, 28-OC*H*_3_), 3.82 (s, 3H, 4″-OC*H*_3_), 4.586–4.593 (m, 1H, H-29), 4.61 (dd, *J* = 9.2 and 12.1 Hz, 1H, H-2′), 4.70 (d, *J* = 2.3 Hz, 1H, H-29), 4.75 (dd, *J* = 4.1 and 12.1 Hz, 1H, H-2′), 6.85 (d, *J* = 8.7 Hz, 2H, H-3″,5″), 7.08 (d, *J* = 8.7 Hz, 2H, H-2″,6″) ppm. ^13^C NMR (125.77 MHz, CDCl_3_): δ = 14.6 and 15.2 (C-26,27), 18.6 (C-25), 19.3 and 28.6 (C-23,24), 19.5 (C-30), 20.1, 21.8, 25.5, 29.6, 30.6, 32.0, 32.7, 36.9, 37.1, 38.3, 40.6, 42.4, 42.8 (C-2), 44.1 (C-1′), 46.9, 47.5, 48.3, 49.3, 50.0, 51.3 (28-O*C*H_3_), 52.4, 55.2 (4″-O*C*H_3_), 56.5, 79.2 (C-2′), 109.6 (C-29), 114.3 (C-3″,5″), 129.1 (C-2″,6″), 129.5 (C-1″), 150.6 (C-20), 158.9 (C-4″), 176.6 (C-28), 219.3 (C-3) ppm. HRMS-ESI *m/z* calcd for [C_40_H_57_NO_6_ + H]^+^: 648.4264, found: 648.4261.

*Methyl 2-[1-(4-fluorophenyl)-2-nitroethyl]betulonate *(**4c**)*.* White solid (74 mg, 73% yield). Mp 244–247 °C. ^1^H NMR (300.13 MHz, CDCl_3_): δ = 0.65 (s, 3H, H-25), 0.79–2.29 (m, 22H), 0.86 and 0.94 (2 s, 6H, H-26,27), 1.05 and 1.12 (2 s, 6H, H-23,24), 1.66 (s, 3H, H-30), 2.92–2.99 (m, 1H), 3.20 (q, *J* = 10.0 Hz, 1H, H-2), 3.60 (dt, *J* = 4.5 and 9.3 Hz, 1H, H-1′), 3.65 (s, 3H, 28-OC*H*_3_), 4.59 (br s, 1H, H-29), 4.64 (dd, *J* = 9.3 and 12.5 Hz, 1H, H-2′), 4.70 (d, *J* = 2.2 Hz, 1H, H-29), 4.76 (dd, *J* = 4.5 and 12.5 Hz, 1H, H-2′), 7.03 (t, *J* = 8.6 Hz, 2H, H-3″,5″), 7.15 (dd, *J* = 5.4 and 8.6 Hz, 2H, H-2″,6″) ppm. ^13^C NMR (75.47 MHz, CDCl_3_): δ = 14.6 and 15.2 (C-26,27), 18.6 (C-25), 19.3 and 28.6 (C-23,24), 19.5 (C-30), 20.0, 21.9, 25.4, 29.6, 30.6, 32.0, 32.7, 36.9, 37.1, 38.3, 40.6, 42.4, 42.8 (C-2), 44.1 (C-1′), 46.9, 47.5, 48.3, 49.3, 50.0, 51.3 (28-O*C*H_3_), 52.4, 56.5, 78.9 (C-2′), 109.6 (C-29), 116.0 (d, *J* = 21.4 Hz, C-3″,5″), 129.7 (d, *J* = 8.1 Hz, C-2″,6″), 133.4 (d, *J* = 3.3 Hz, C-1″), 150.5 (C-20), 162.1 (d, *J* = 246.1 Hz, C-4″), 176.6 (C-28), 219.0 (C-3) ppm. HRMS-ESI *m/z* calcd for [C_39_H_54_FNO_5_ + Na]^+^: 658.3884, found: 658.3895.

### 3.4. Synthesis of 1,2-Oxazine-Fused BoOMe Compounds ***5a-c***

Zn powder (1.0 mmol, 65 mg) and NH_4_OAc (1.0 mmol, 77 mg) were added to a solution of Michael adducts **4a-c** (0.10 mmol) in a mixture of DCM/MeOH (1:1) (2 mL). The reaction was stirred at room temperature for 5 h. After this period, the inorganic salts were removed by filtration, and the solvent was evaporated to dryness. The obtained residue was dissolved in DCM and then purified by preparative TLC using a mixture of Hex/EtOAc (2:1) as eluent. Two products were obtained, being the desired 1,2-oxazine-fused BoOMe compounds **5a-c** correspondent to the fraction of the lower *R_f_* value.

*4-Phenyl-1,2-oxazine-fused BoOMe compound ***5a****. Colorless oil (21.1 mg, 36% yield). ^1^H NMR (300.13 MHz, CDCl_3_): δ = 0.79–2.29 (m, 23H), 0.83 (s, 3H, H-25), 0.93 and 0.97 (2 s, 6H, H-26,27), 1.28 and 1.33 (2 s, 6H, H-23,24), 1.69 (s, 3H, H-30), 2.97–3.05 (m, 1H), 3.25 (t, *J* = 8.3 Hz, 1H, H-4′), 3.67 (s, 3H, 28-OC*H*_3_), 4.09 (dd, *J* = 8.3 and 15.2 Hz, 1H, H-3′), 4.29 (dd, *J* = 8.3 and 15.2 Hz, 1H, H-3′), 4.61 (dd, *J* = 1.4 and 2.4 Hz, 1H, H-29), 4.74 (d, *J* = 2.4 Hz, 1H, H-29), 7.22–7.40 (m, 5H, H-2″,3″,4″,5″,6″) ppm. ^13^C NMR (75.47 MHz, CDCl_3_): δ = 14.6 and 15.2 (C-26,27), 17.7 (C-25), 19.4 (C-30), 19.8, 21.8, 23.4 and 30.8 (C-23,24), 25.6, 29.6, 32.0, 32.9, 36.9, 37.7, 38.0, 38.4, 40.5, 42.5, 43.0, 46.9, 49.3, 49.8, 49.9, 51.3 (28-O*C*H_3_), 51.8 (C-4′), 54.7, 56.5, 63.1 (C-3′), 92.9 (C-2), 109.6 (C-29), 127.1 (C-4″), 128.0 and 128.7 (C-2″,6″ and C-3″,5″), 136.7 (C-1″), 150.6 (C-20), 176.6 (C-28), 181.5 (C-3) ppm. HRMS-ESI *m/z* calcd for [C_39_H_55_NO_3_ + H]^+^: 586.4260, found: 586.4268.

*4-(4-Methoxyphenyl)-1,2-oxazine-fused BoOMe compound ***5b****. Colorless oil (15.4 mg, 25% yield). ^1^H NMR (300.13 MHz, CDCl_3_): δ = 0.82–2.30 (m, 23H), 0.82 (s, 3H, H-25), 0.93 and 0.97 (2 s, 6H, H-26,27), 1.28 and 1.33 (2 s, 6H, H-23,24), 1.69 (s, 3H, H-30), 2.97–3.05 (m, 1H), 3.19 (t, *J* = 8.3 Hz, 1H, H-4′), 3.67 (s, 3H, 28-OC*H*_3_), 3.81 (s, 3H, 4″-OC*H*_3_), 4.05 (dd, *J* = 8.3 and 15.1 Hz, 1H, H-3′), 4.26 (dd, *J* = 8.3 and 15.1 Hz, 1H, H-3′), 4.61 (br s, 1H, H-29), 4.74 (d, *J* = 1.9 Hz, 1H, H-29), 6.91 (d, *J* = 8.7 Hz, 2H, H-3″,5″), 7.30 (d, *J* = 8.7 Hz, 2H, H-2″,6″) ppm. ^13^C NMR (75.47 MHz, CDCl_3_): δ = 14.6 and 15.2 (C-26,27), 17.7 (C-25), 19.4 (C-30), 19.8, 21.8, 23.4 and 30.8 (C-23,24), 25.6, 29.6, 30.5, 32.0, 32.8, 36.9, 37.8, 38.0, 38.3, 40.5, 42.5, 46.9, 49.3, 49.8, 49.9, 51.3 (28-O*C*H_3_), 51.6, 54.0 (C-4′), 55.3 (4″-O*C*H_3_), 56.5, 63.1 (C-3′), 92.7 (C-2), 109.6 (C-29), 114.2 (C-3″,5″), 128.2 (C-1″), 129.0 (C-2″,6″), 150.6 (C-20), 158.6 (C-4″), 176.6 (C-28), 182.1 (C-3) ppm. HRMS-ESI *m/z* calcd for [C_40_H_57_NO_4_ + H]^+^: 616.4366, found: 616.4346.

*4-(4-Fluorophenyl)-1,2-oxazine-fused BoOMe compound ***5c***.* Colorless oil (13.9 mg, 23% yield). ^1^H NMR (300.13 MHz, CDCl_3_): δ = 0.80–2.30 (m, 23H), 0.83 (s, 3H, H-25), 0.93 and 0.97 (2 s, 6H, H-26,27), 1.28 and 1.33 (2 s, 6H, H-23,24), 1.69 (s, 3H, H-30), 2.96–3.05 (m, 1H), 3.19 (t, *J* = 8.3 Hz, 1H, H-4′), 3.67 (s, 3H, 28-OC*H*_3_), 4.04 (dd, *J* = 8.3 and 15.1 Hz, 1H, H-3′), 4.28 (dd, *J* = 8.3 and 15.1 Hz, 1H, H-3′), 4.60–4.61 (m, 1H, H-29), 4.74 (d, *J* = 2.3 Hz, 1H, H-29), 7.05 (t, *J* = 8.7 Hz, 2H, H-3″,5″), 7.35 (dd, *J* = 5.4 and 8.7 Hz, 2H, H-2″,6″) ppm. ^13^C NMR (75.47 MHz, CDCl_3_): δ = 14.6 and 15.2 (C-26,27), 17.6 (C-25), 19.4 (C-30), 19.8, 21.8, 23.4 and 30.5 (C-23,24), 25.6, 29.7, 30.9, 32.0, 32.8, 36.9, 37.7, 38.1, 38.3, 40.5, 42.5, 46.9, 49.3, 49.90, 49.94, 51.3 (28-O*C*H_3_), 51.5, 54.3 (C-4′), 56.5, 63.5 (C-3′), 92.6 (C-2), 109.6 (C-29), 115.5 (d, *J* = 21.0 Hz, C-3″,5″), 129.6 (d, *J* = 7.8 Hz, C-2″,6″), 132.3 (d, *J* = 3.3 Hz, C-1″), 150.6 (C-20), 161.8 (d, *J* = 245.6 Hz, C-4″), 176.6 (C-28), 181.4 (C-3) ppm. HRMS-ESI *m/z* calcd for [C_39_H_54_FNO_3_ + H]^+^: 604.4166, found: 604.4174.

### 3.5. Synthesis of the Pyran-Fused BoOMe Compound ***6***

Methyl (*E*)-2-(4-methoxybenzylidene)betulonate **3b** (58.7 mg, 0.100 mmol), malononitrile (33.0 mg, 0.500 mmol), DBU (10 mol%) and EtOH (2 mL) were mixed in a CEM MW glass reactor. The resulting mixture was heated at 100 °C for 30 min under MW irradiation. After that period, the solvent was removed under reduced pressure, and the crude product dissolved in DCM and purified by preparative TLC, using a mixture of Hex/EtOAc (10:1) as eluent. Compound **6** was obtained as an inseparable mixture of two diastereomers **d_M_-6** and **d_m_-6** (67:33). White solid (41.8 mg, 64% yield). ^1^H NMR (300.13 MHz, CDCl_3_): **d_M_-6:** δ = 0.56–3.00 (m, 41H), 3.64 (s, 1H, H-4′), 3.66 (s, 3H, 28-OC*H*_3_), 3.80 (s, 3H, 4″-OC*H*_3_), 4.35 (br s, 2H, N*H*_2_), 4.56–4.57 (m, 1H, H-29), 4.70–4.71 (m, 1H, H-29), 6.85 (d, *J* = 8.6 Hz, 2H, H-3″,5″), 7.08 (d, *J* = 8.6 Hz, 2H, H-2″,6″) ppm; **d_m_-6**: δ = 0.56–3.00 (m, 41H), 3.63 (s, 1H, H-4′), 3.65 (s, 3H, 28-OC*H*_3_), 3.79 (s, 3H, 4″-OC*H*_3_), 4.35 (br s, 2H, N*H*_2_), 4.56–4.57 (m, 1H, H-29), 4.70–4.71 (m, 1H, H-29), 6.81 (d, *J* = 8.7 Hz, 2H, H-3″,5″), 7.04 (d, *J* = 8.7 Hz, 2H, H-2″,6″) ppm. ^13^C NMR (75.47 MHz, CDCl_3_): **d_M_-6**: δ = 14.67, 15.57, 16.3, 19.2, 19.3, 25.4, 28.3, 29.4, 30.54, 32.1, 33.2, 36.0, 36.4, 36.9, 38.3, 40.49, 41.83, 42.39 (C-4′), 43.3, 44.0, 46.9, 48.7, 49.3, 51.3 (28-O*C*H_3_), 52.7, 55.24 (4″-O*C*H_3_), 56.52, 60.92 (C-3′), 106.4 (C-2), 109.59 (C-29), 114.1 (C-3″,5″), 120.3 (*C*N), 128.7 (C-2″,6″), 135.6 (C-1″), 147.91 (C-3), 150.6 (C-20), 158.6 (C-4″), 159.0 (C-2′), 176.6 (C-28) ppm; **d_m_-6**: δ = 14.63, 15.59, 16.3, 19.2, 19.3, 25.4, 27.7, 29.6, 30.49, 31.6, 33.3, 36.0, 36.3, 36.9, 38.3, 40.52, 41.76, 42.36 (C-4′), 43.3, 44.0, 46.9, 48.9, 49.3, 51.3 (28-O*C*H_3_), 53.0, 55.22 (4″-O*C*H_3_), 56.49, 60.88 (C-3′), 107.1 (C-2), 109.65 (C-29), 113.9 (C-3″,5″), 120.4 (*C*N), 129.0 (C-2″,6″), 135.4 (C-1″), 147.88 (C-3), 150.5 (C-20), 159.0 (C-2′), 159.4 (C-4″), 176.6 (C-28) ppm. HRMS-ESI *m/z* calcd for [C_42_H_56_N_2_O_4_ + H]^+^: 653.4318, found: 653.4295.

### 3.6. Synthesis of the Diarylpyridine-Fused BoOMe Compound ***8***

Methyl (*E*)-2-(4-methoxybenzylidene)betulonate (**3b**) (70 mg, 0.12 mmol) was added to a stirring solution of the pyridinium salt **7** (66 mg, 0.24 mmol) and NH_4_OAc (185 mg, 2.4 mmol) in AcOH (4 mL). The resulting reaction mixture was kept under reflux for 24 h. After that period, the solvent was removed under reduced pressure, and the crude residue was dissolved in DCM and purified by preparative TLC, using a mixture of Hex/EtOAc (10:1) as eluent, affording the desired diarylpyridine-fused BoOMe compound **8** as a colorless oil (21 mg, 25% yield). ^1^H NMR (500.13 MHz, CDCl_3_): δ = 0.74 (s, 3H, H-25), 0.85–2.26 (m, 21H), 0.96 and 1.00 (2 s, 6H, H-26,27), 1.40 and 1.43 (2 s, 6H, H-23,24), 1.70 (s, 3H, H-30), 2.79 (d, *J* = 16.0 Hz, 1H), 2.97–3.02 (m, 1H), 3.67 (s, 3H, 28-OC*H*_3_), 3.90 (s, 3H, 4″-OC*H*_3_), 4.61–4.62 (m, 1H, H-29), 4.74 (d, *J* = 2.4 Hz, 1H, H-29), 6.99 (d, *J* = 8.7 Hz, 2H, H-3″,5″), 7.23 (d, *J* = 8.7 Hz, 2H, H-2″,6″), 7.35–7.46 (m, 3H, H-3‴,4‴,5‴), 7.39 (s, 1H, H-3′), 8.08–8.12 (m, 2H, H-2‴,6‴) ppm. ^13^C NMR (125.77 MHz, CDCl_3_): δ = 14.7 and 15.6 (C-26,27), 15.6 (C-25), 19.5 (C-30), 20.4, 21.4, 22.7, 24.7 and 32.4 (C-23,24), 25.6, 29.7, 30.7, 31.9, 32.1, 33.4, 36.3, 37.0, 38.3, 40.2, 40.6, 42.5, 43.3, 46.9, 48.9, 49.4, 51.3 (28-O*C*H_3_), 53.3, 55.3 (4″-O*C*H_3_), 56.6, 109.5 (C-29), 113.8 (C-3″,5″), 118.4 (C-3′), 125.8 (C-2), 126.5 (C-2‴,6‴), 128.5 (C-3‴,4‴,5‴), 129.9 (C-2″,6″), 132.6 (C-1″), 139.3 (C-1‴), 150.2 (C-4′), 150.8 (C-20), 153.1 (C-2′), 159.0 (C-4″), 163.8 (C-3), 176.7 (C-28) ppm. HRMS-ESI *m/z* calcd for [C_47_H_59_NO_3_ + H]^+^: 686.4573, found: 686.4579.

### 3.7. Synthesis of the 1,2,3-Triazole–BoOMe Compound ***11***

NaN_3_ (19.3 mg, 0.297 mmol) was added to a solution of methyl (*E*)-2-[3-(4-methoxyphenyl)prop-2-yn-1-ylidene]betulonate (**10**) (36 mg, 0.059 mmol) in dry DMF (3 mL) in a CEM MW glass reactor. The reaction mixture was heated at 160 °C under MW irradiation for 30 min. After this period, it was poured onto ice and H_2_O, and the pH was adjusted to 4 with diluted HCl. The obtained precipitate was filtered and dissolved in EtOAc, and the resulting solution was dried over anhydrous Na_2_SO_4_. The solvent was evaporated to dryness, affording the desired 1,2,3-triazole–BoOMe compound **11** as a beige solid (38 mg, quantitative yield). Mp 174–177 °C. ^1^H NMR (300.13 MHz, CDCl_3_): δ = 0.67–2.39 (m, 21H), 0.82 (s, 3H, H-25), 0.97 and 1.02 (2 s, 6H, H-26,27), 1.10 and 1.16 (2 s, 6H, H-23,24), 1.72 (s, 3H, H-30), 2.99–3.08 (m, 1H), 3.45 (dd, *J* = 1.8 and 18.1 Hz, 1H), 3.68 (s, 3H, 28-OC*H*_3_), 3.85 (s, 3H, 4″-OC*H*_3_), 4.64–4.65 (m, 1H, H-29), 4.77 (d, *J* = 2.0 Hz, 1H, H-29), 7.00 (d, *J* = 8.8 Hz, 2H, H-3″,5″), 7.48–7.49 (m, 1H, –C*H*=), 7.54 (d, *J* = 8.8 Hz, 2H, H-2″,6″) ppm. ^13^C NMR (75.47 MHz, CDCl_3_): δ = 14.7 and 15.4 (C-26,27), 16.1 (C-25), 19.5 (C-30), 20.4, 21.7, 22.3 and 29.5 (C-23,24), 25.7, 29.7, 30.6, 32.1, 33.1, 36.1, 37.0, 38.4, 40.5, 42.5, 45.1, 45.4, 46.9, 48.3, 49.4, 51.4 (28-O*C*H_3_), 52.8, 55.4 (4″-O*C*H_3_), 56.6, 109.6 (C-29), 114.5 (C-3″,5″), 121.5 (C-1″), 123.1 (–*C*H=), 129.8 (C-2′,6′), 137.2 (C-2), 139.9 (C-4′), 146.4(C-5′), 150.7 (C-20), 160.3 (C-4″), 176.7 (C-28), 208.1 (C-3) ppm. HRMS-ESI *m/z* calcd for [C_41_H_55_N_3_O_4_ + H]^+^: 654.4271, found: 654.4248.

## 4. Conclusions

To conclude, the novel compounds based on a BA-derived skeleton incorporating 1,2-oxazine, 2-amino-3-cyano-4*H*-pyran, diarylpyridine and 1,2,3-triazole moieties were synthesized. To the best of our knowledge, 1,2-oxazines and this specific type of pyran were installed for the first time in the BA skeleton. Remarkably, during the three-step reaction process to prepare the 1,2-oxazine-fused BoOMe compounds, the Michael addition of nitromethane to methyl (*E*)-2-benzylidenebetulonate intermediates occurred in a diastereoselective manner, which led to the observation of just one diastereomer out of four possible ones. Although the employed methodologies are already known from the literature, they had never been applied to this triterpenic acid before. In addition, they allow a molecular diversification, leading to the creation of large libraries of added-value BA-based compounds with potential biological applications.

## Data Availability

Not applicable.

## Supplementary Materials

The following supporting information can be downloaded at: https://www.mdpi.com/article/10.3390/molecules27154904/s1, Figures S1–S6, S8–S13, S15, S16 and S18–S25: ^1^H and ^13^C NMR spectra of all synthesized compounds; Figure S14: ATR-FTIR spectrum of compound **5b**; Figures S7 and S17: NOESY spectra of compounds **4b** and **5c**, respectively.

## Sample Availability

Samples of the compounds are not available from the authors.
