# Supplementary material for: Decoration of A-Ring of a Lupane-Type Triterpenoid with Different Oxygen and Nitrogen Heterocycles
Source: Molecules. 2022 Jul 31;27(15):4904. doi: 10.3390/molecules27154904 (PMC9370315; doi:10.3390/molecules27154904)
Supplement: Supplementary file 1 [file molecules-27-04904-s001.zip › molecules-1724500-supplementary.pdf]

Decoration of A-ring of a lupane-type triterpenoid with different oxygen and nitrogen heterocycles

Joana L. C. Sousa [1,*,†], Hélio M. T. Albuquerque [1,†], Armando J. D. Silvestre [2] and Artur M. S. Silva [1,*]

[1] LAQV-REQUIMTE, Department of Chemistry, University of Aveiro, 3810-193 Aveiro, Portugal; helio.albuquerque@ua.pt (H.M.T.A.)
[2] CICECO – Aveiro Institute of Materials, Department of Chemistry, University of Aveiro, 3810-193 Aveiro, Portugal; armsil@ua.pt (A.J.D.S.)
* Correspondence: joanasousa@ua.pt (J.L.C.S.); artur.silva@ua.pt (A.M.S.S.); Tel.: +351-234-370-714 (A.M.S.S.)
† J.L.C.S. and H.M.T.A. contributed equally to this work.

Contents

Figure S1. ^{1}H NMR spectrum of methyl (*E*)-2-(4-fluorobenzylidene)betulonate (**3c**) (300.13 MHz, $CDCl_3$). 3
Figure S2. ^{13}C NMR spectrum of methyl (*E*)-2-(4-fluorobenzylidene)betulonate (**3c**) (75.47 MHz, $CDCl_3$)........ 3
Figure S3. ^{1}H NMR spectrum of methyl 2-(1-phenyl-2-nitroethyl)betulonate (**4a**) (500.13 MHz, $CDCl_3$)........ 4
Figure S4. ^{13}C NMR spectrum of methyl 2-(1-phenyl-2-nitroethyl)betulonate (**4a**) (125.77 MHz, $CDCl_3$)........ 4
Figure S5. ^{1}H NMR spectrum of methyl 2-[1-(4-methoxyphenyl)-2-nitroethyl]betulonate (**4b**) (500.13 MHz, $CDCl_3$). 5
Figure S6. ^{13}C NMR spectrum of methyl 2-[1-(4-methoxyphenyl)-2-nitroethyl]betulonate (**4b**) (125.77 MHz, $CDCl_3$). 5
Figure S7. NOESY spectrum of the methyl 2-[1-(4-methoxyphenyl)-2-nitroethyl]betulonate (**4b**) (500.13 MHz, $CDCl_3$). 6
Figure S8. ^{1}H NMR spectrum of methyl 2-[1-(4-fluorophenyl)-2-nitroethyl]betulonate (**4c**) (300.13 MHz, $CDCl_3$). 7
Figure S9. ^{13}C NMR spectrum of methyl 2-[1-(4-fluorophenyl)-2-nitroethyl]betulonate (**4c**) (75.47 MHz, $CDCl_3$). 7
Figure S10. ^{1}H NMR spectrum of the 4-phenyl-1,2-oxazine-fused BoOMe compound **5a** (300.13 MHz, $CDCl_3$). 8
Figure S11. ^{13}C NMR spectrum of the 4-phenyl-1,2-oxazine-fused BoOMe compound **5a** (75.47 MHz, $CDCl_3$). 8
Figure S12. ^{1}H NMR spectrum of the 4-(4-methoxyphenyl)-1,2-oxazine-fused BoOMe compound **5b** (300.13 MHz, $CDCl_3$)........ 9
Figure S13. ^{13}C NMR spectrum of the 4-(4-methoxyphenyl)-1,2-oxazine-fused BoOMe compound **5b** (75.47 MHz, $CDCl_3$)........ 9
Figure S14. ATR-FTIR spectrum of the 4-(4-methoxyphenyl)-1,2-oxazine-fused BoOMe compound **5b** (FTIR Bruker Tensor 27, with ATR Golden Gate accessory, Diamond (Specac), Absorbance mode, resolution 4 cm^{-1}, 256 scans, 4000-500 cm^{-1}). 10

Figure S15. ^{1}H NMR spectrum of the 4-(4-fluorophenyl)-1,2-oxazine-fused BoOMe compound **5c** (300.13 MHz, $CDCl_3$). 11
Figure S16. ^{13}C NMR spectrum of the 4-(4-fluorophenyl)-1,2-oxazine-fused BoOMe compound **5c** (75.47 MHz, $CDCl_3$). 11
Figure S17. NOESY spectrum of the 4-(4-fluorophenyl)-1,2-oxazine-fused BoOMe compound **5c** (300.13 MHz, $CDCl_3$). 12
Figure S18. ^{1}H NMR spectrum of the pyran-fused BoOMe compound **6** (300.13 MHz, $CDCl_3$).. 13
Figure S19. ^{13}C NMR spectrum of the pyran-fused BoOMe compound **6** (75.47 MHz, $CDCl_3$)... 13
Figure S20. ^{1}H NMR spectrum of the diarylpyridine-fused BoOMe compound **8** (500.13 MHz, $CDCl_3$). 14
Figure S21. ^{13}C NMR spectrum of the diarylpyridine-fused BoOMe compound **8** (125.77 MHz, $CDCl_3$). 14
Figure S22. ^{1}H NMR spectrum of methyl (*E*)-2-[3-(4-methoxyphenyl)prop-2-yn-1-ylidene]betulonate (**10**) (300.13 MHz, $CDCl_3$). 15
Figure S23. ^{13}C NMR spectrum of methyl (*E*)-2-[3-(4-methoxyphenyl)prop-2-yn-1-ylidene]betulonate (**10**) (75.47 MHz, $CDCl_3$). 15
Figure S24. ^{1}H NMR spectrum of the 1,2,3-triazole–BoOMe compound **11** (300.13 MHz, $CDCl_3$). 16
Figure S25. ^{13}C NMR spectrum of the 1,2,3-triazole–BoOMe compound **11** (75.47 MHz, $CDCl_3$). 16

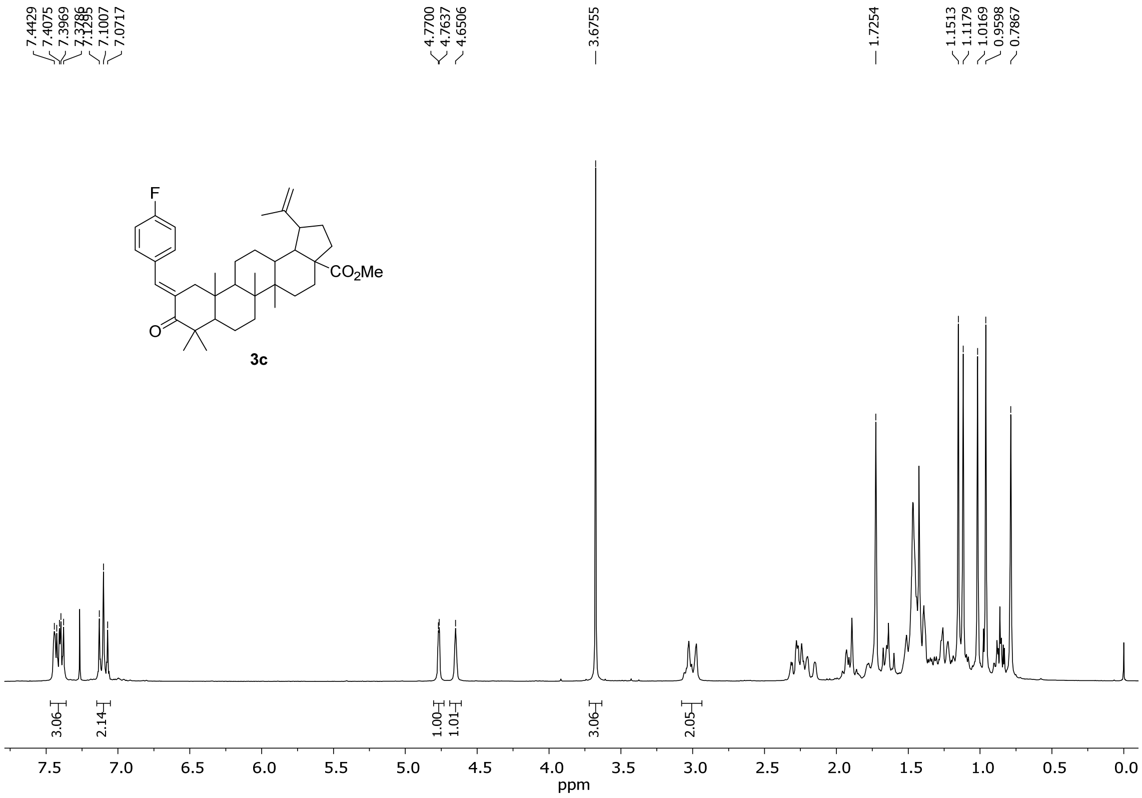

Figure S1. ^{1}H NMR spectrum of methyl (*E*)-2-(4-fluorobenzylidene)betulonate (**3c**) (300.13 MHz, $CDCl_3$).

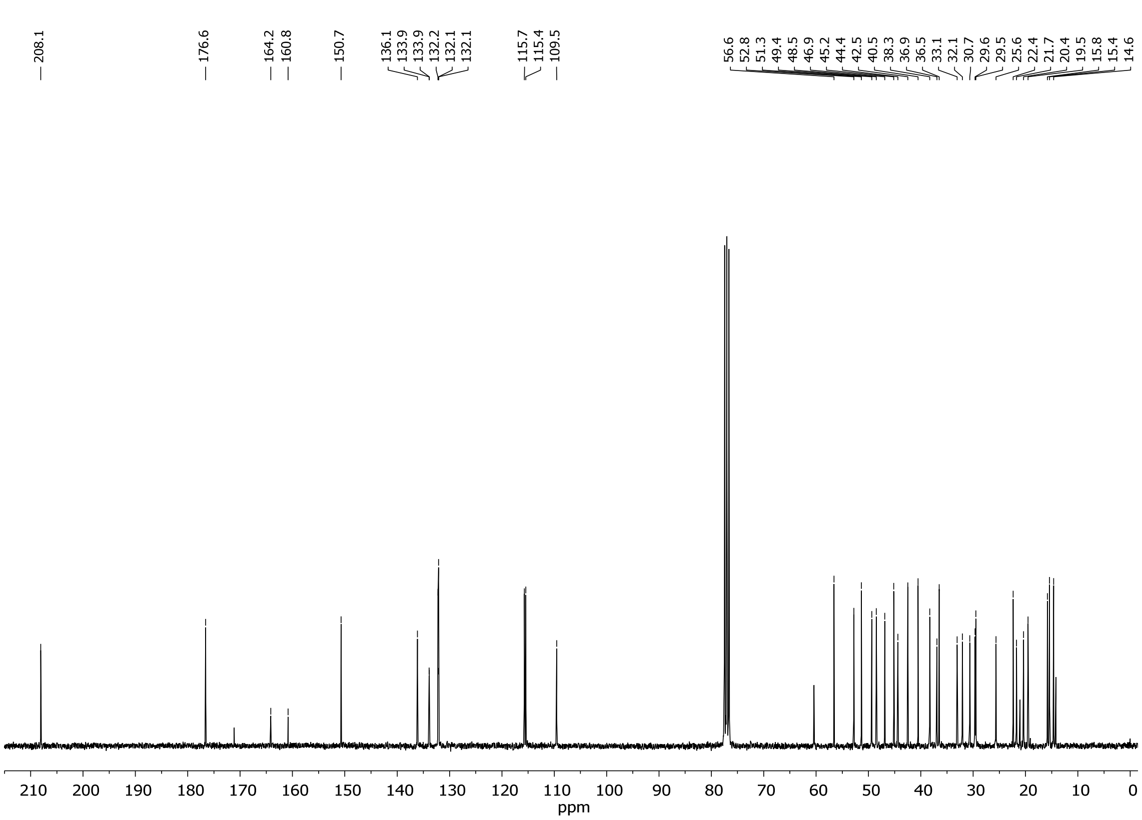

Figure S2. ^{13}C NMR spectrum of methyl (*E*)-2-(4-fluorobenzylidene)betulonate (**3c**) (75.47 MHz, $CDCl_3$).

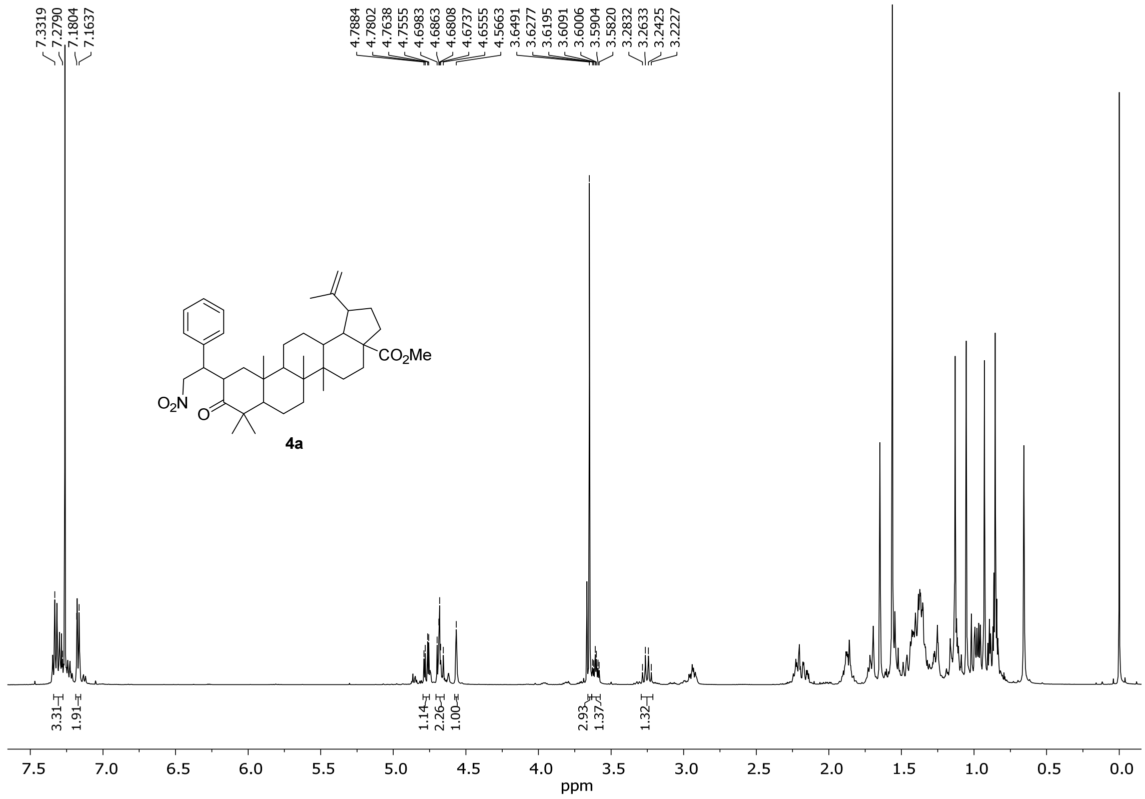

Figure S3. ^{1}H NMR spectrum of methyl 2-(1-phenyl-2-nitroethyl)betulonate (**4a**) (500.13 MHz, $CDCl_3$).

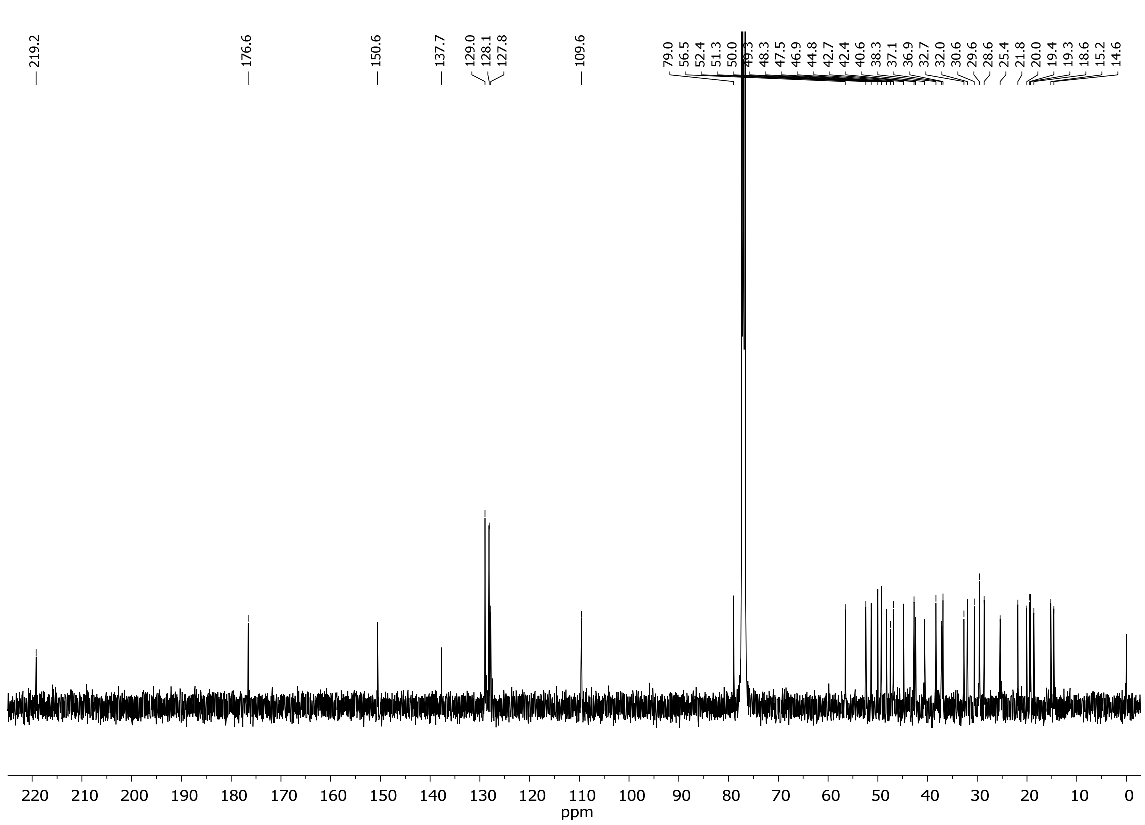

Figure S4. ^{13}C NMR spectrum of methyl 2-(1-phenyl-2-nitroethyl)betulonate (**4a**) (125.77 MHz, $CDCl_3$).

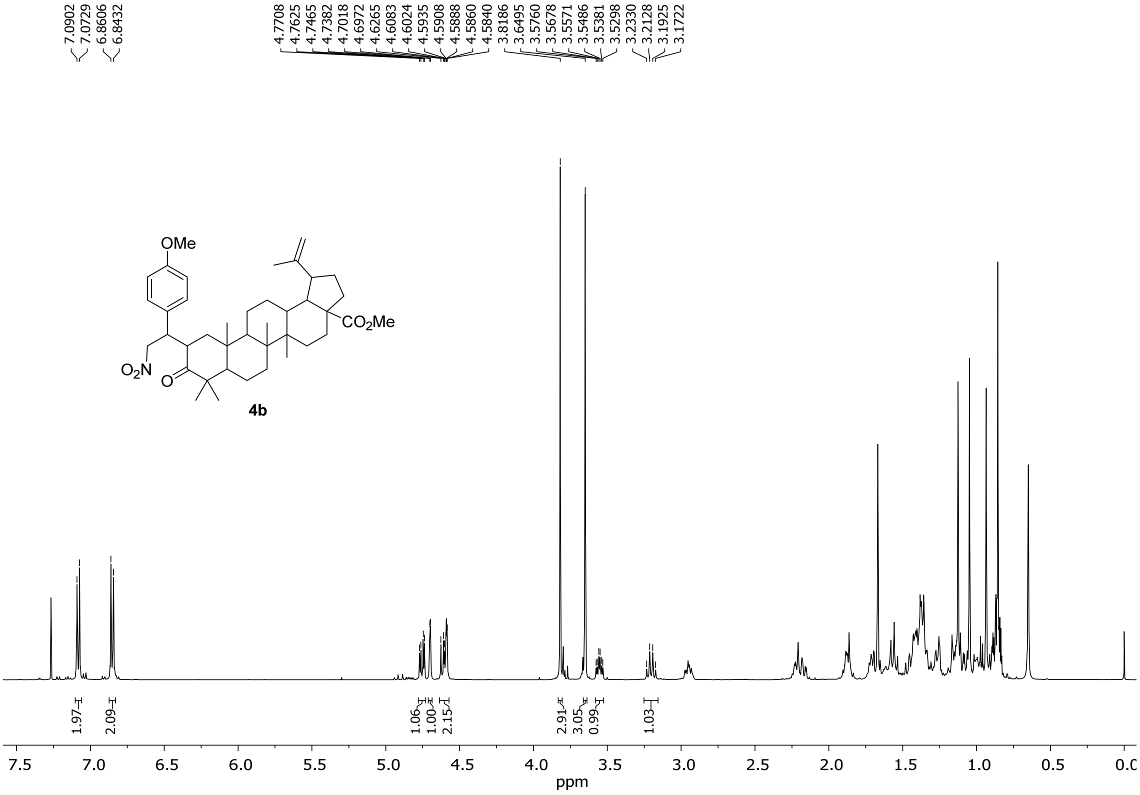

Figure S5. ^{1}H NMR spectrum of methyl 2-[1-(4-methoxyphenyl)-2-nitroethyl]betulonate (**4b**) (500.13 MHz, $CDCl_3$).

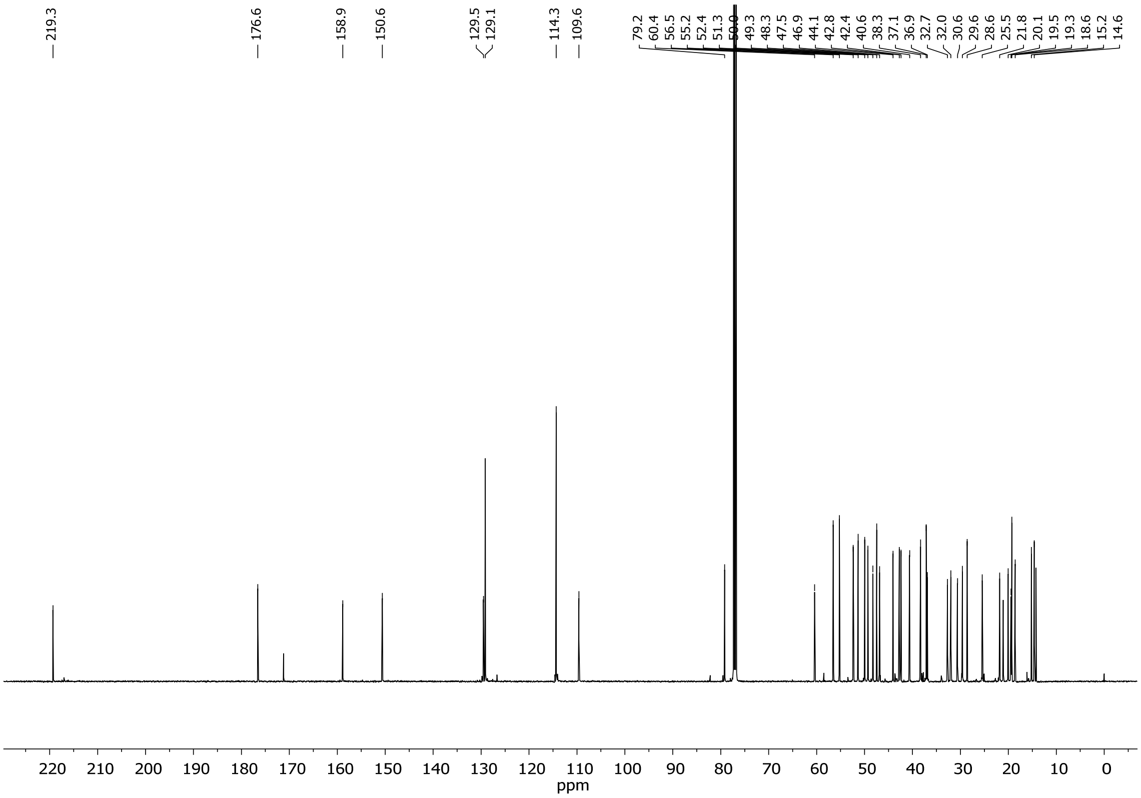

Figure S6. ^{13}C NMR spectrum of methyl 2-[1-(4-methoxyphenyl)-2-nitroethyl]betulonate (**4b**) (125.77 MHz, $CDCl_3$).

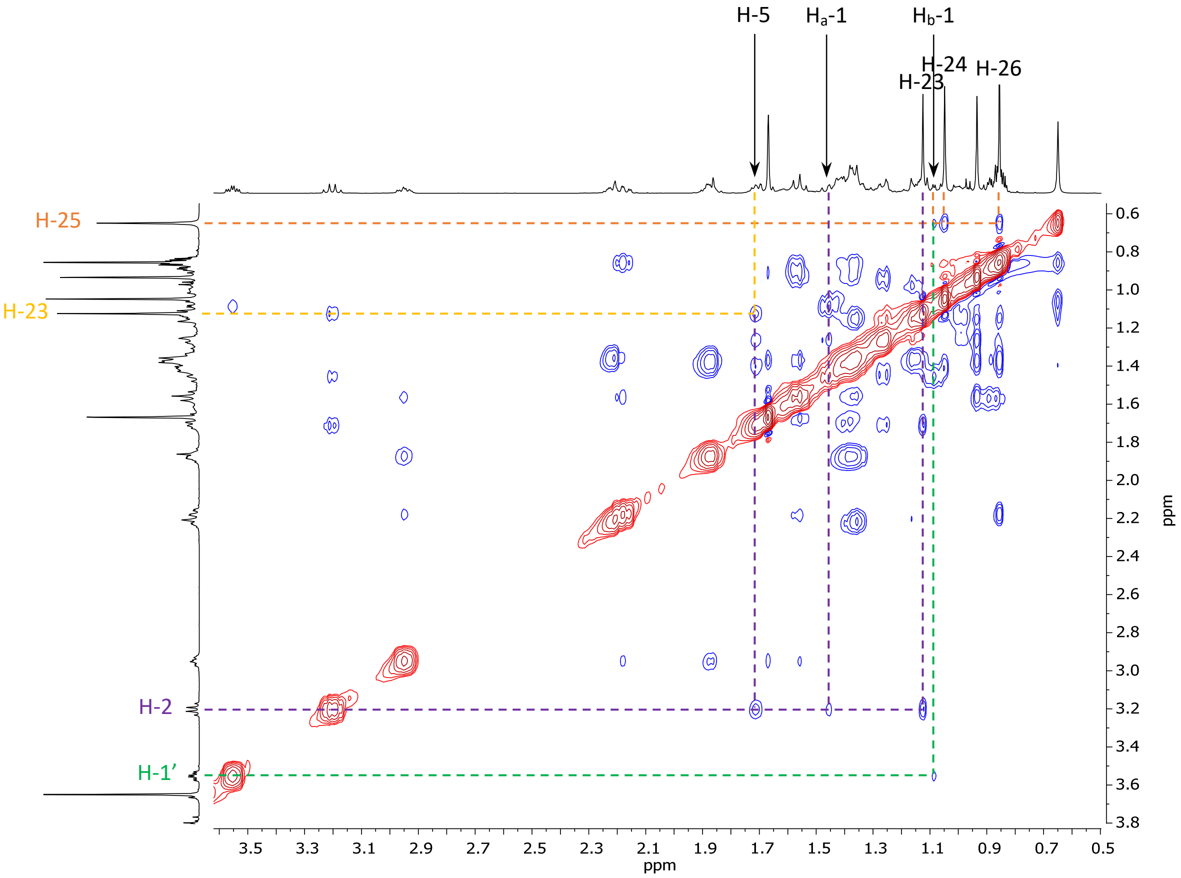

Figure S7. NOESY spectrum of the methyl 2-[1-(4-methoxyphenyl)-2-nitroethyl]betulonate (**4b**) (500.13 MHz, $CDCl_3$).

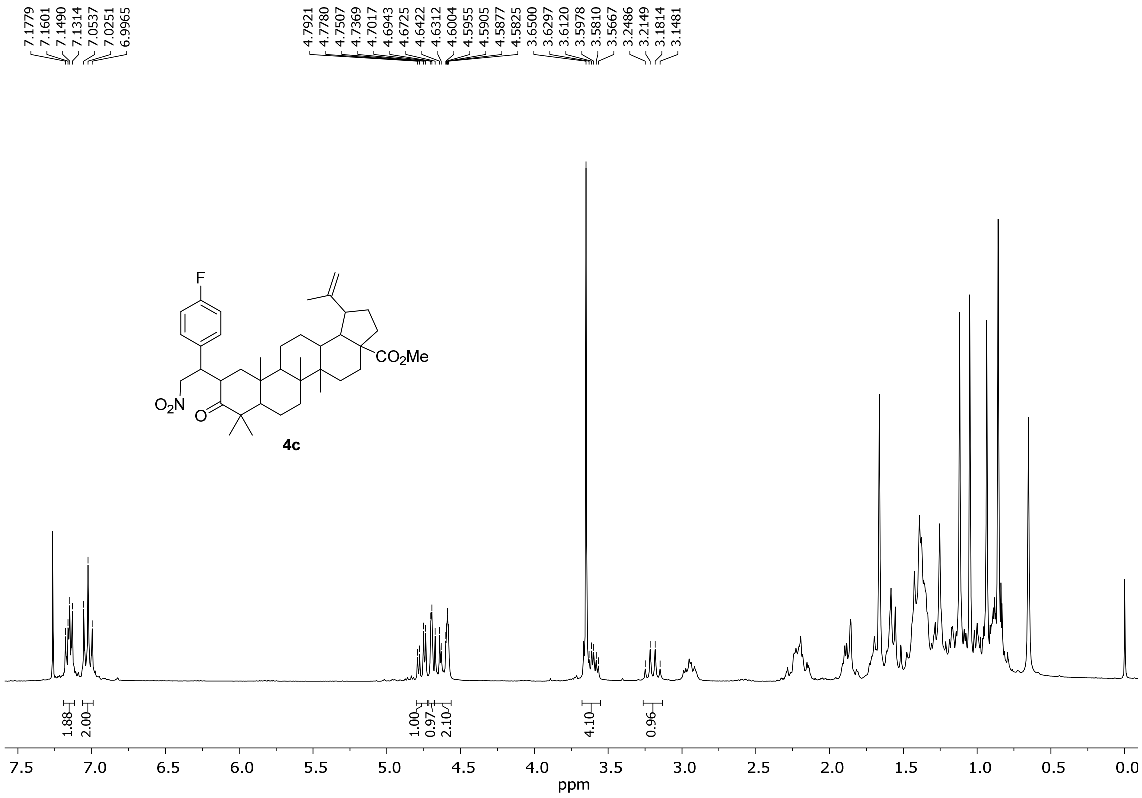

Figure S8. ^{1}H NMR spectrum of methyl 2-[1-(4-fluorophenyl)-2-nitroethyl]betulonate (**4c**) (300.13 MHz, $CDCl_3$).

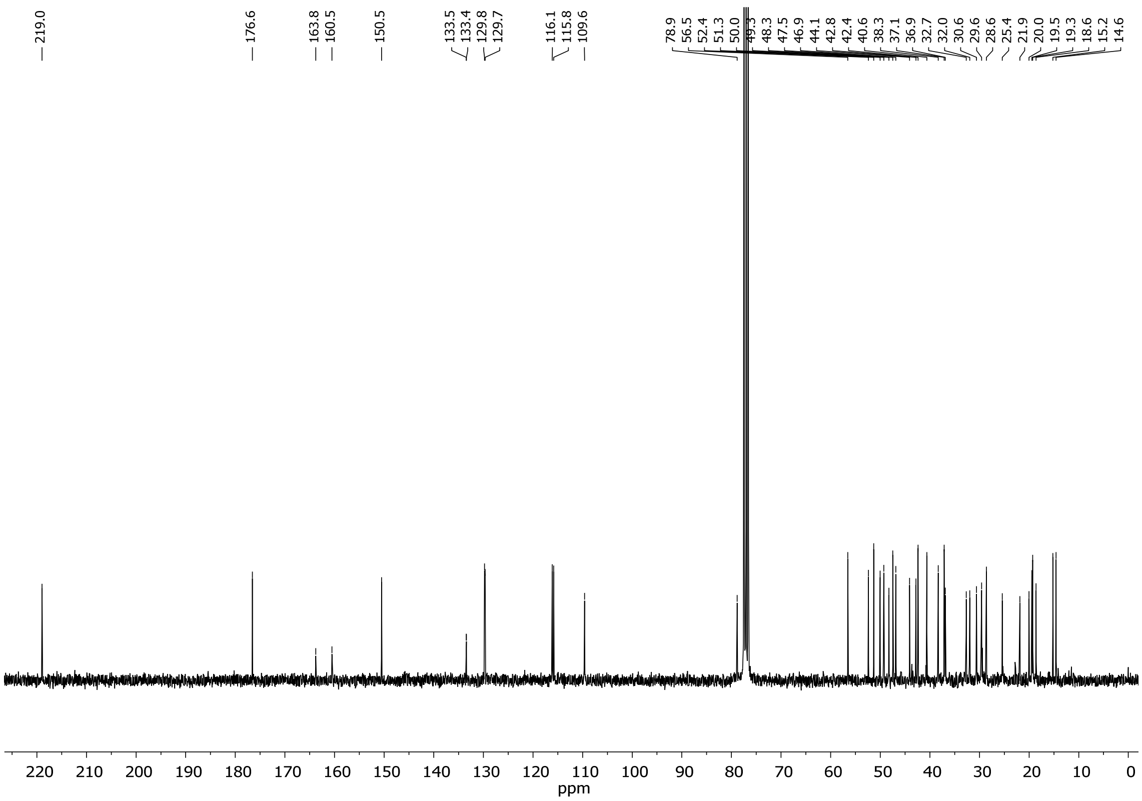

Figure S9. ^{13}C NMR spectrum of methyl 2-[1-(4-fluorophenyl)-2-nitroethyl]betulonate (**4c**) (75.47 MHz, $CDCl_3$).

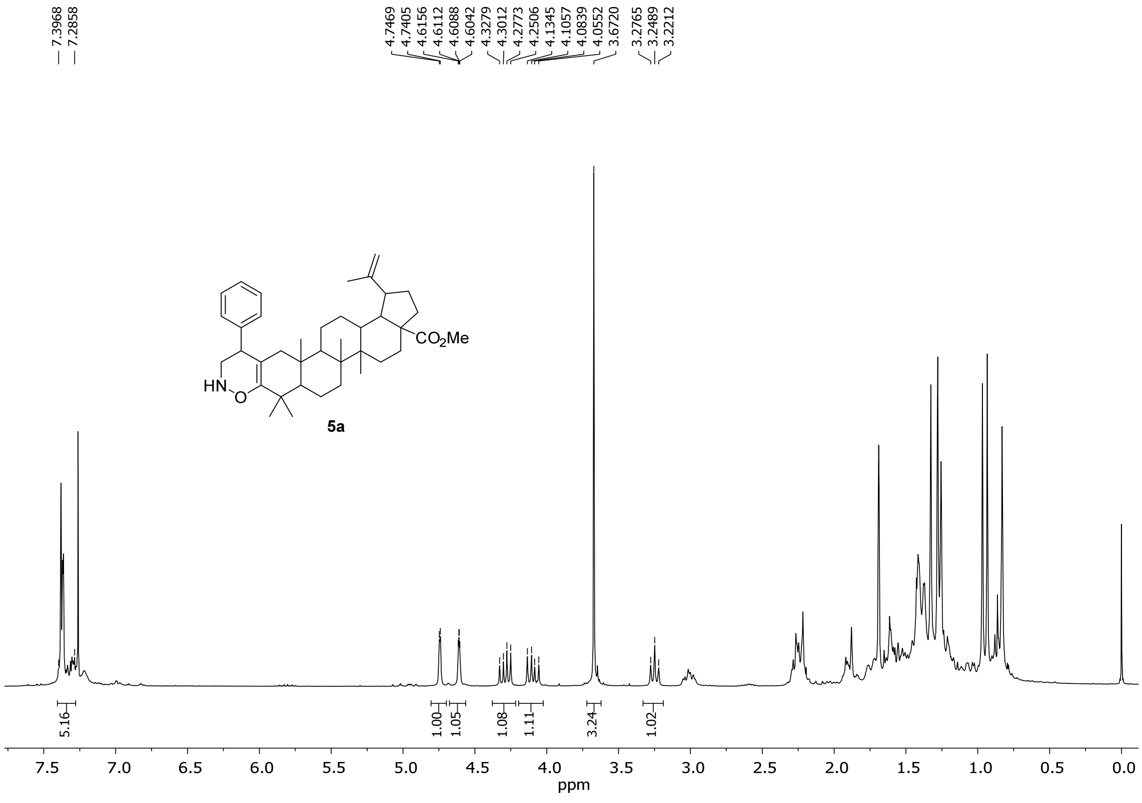

Figure S10. ^{1}H NMR spectrum of the 4-phenyl-1,2-oxazine-fused BoOMe compound **5a** (300.13 MHz, $CDCl_3$).

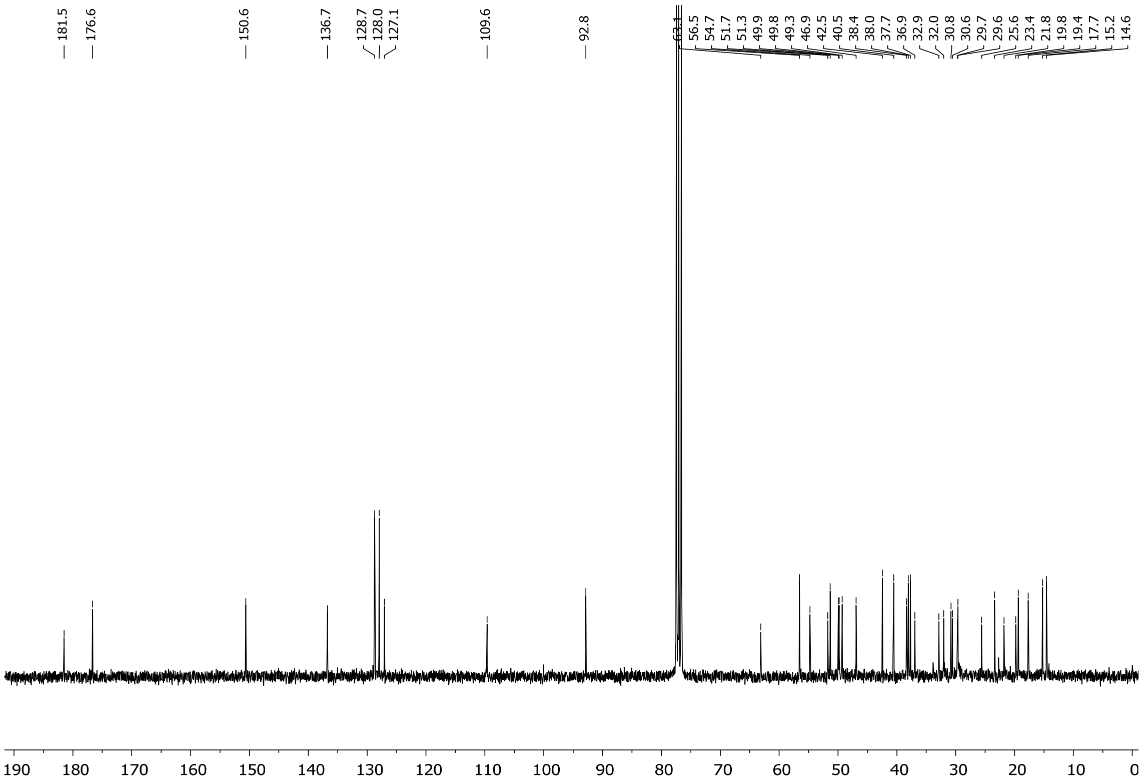

Figure S11. ^{13}C NMR spectrum of the 4-phenyl-1,2-oxazine-fused BoOMe compound **5a** (75.47 MHz, $CDCl_3$).

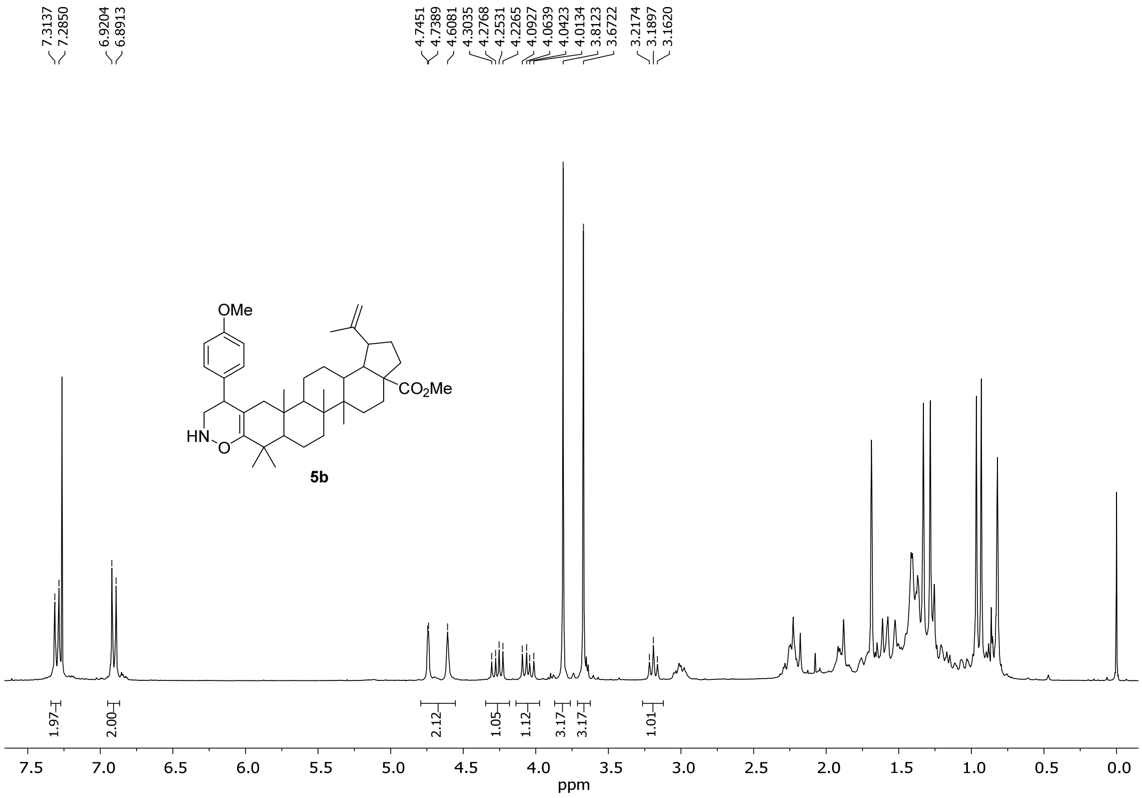

Figure S12. ^{1}H NMR spectrum of the 4-(4-methoxyphenyl)-1,2-oxazine-fused BoOMe compound **5b** (300.13 MHz, $CDCl_3$).

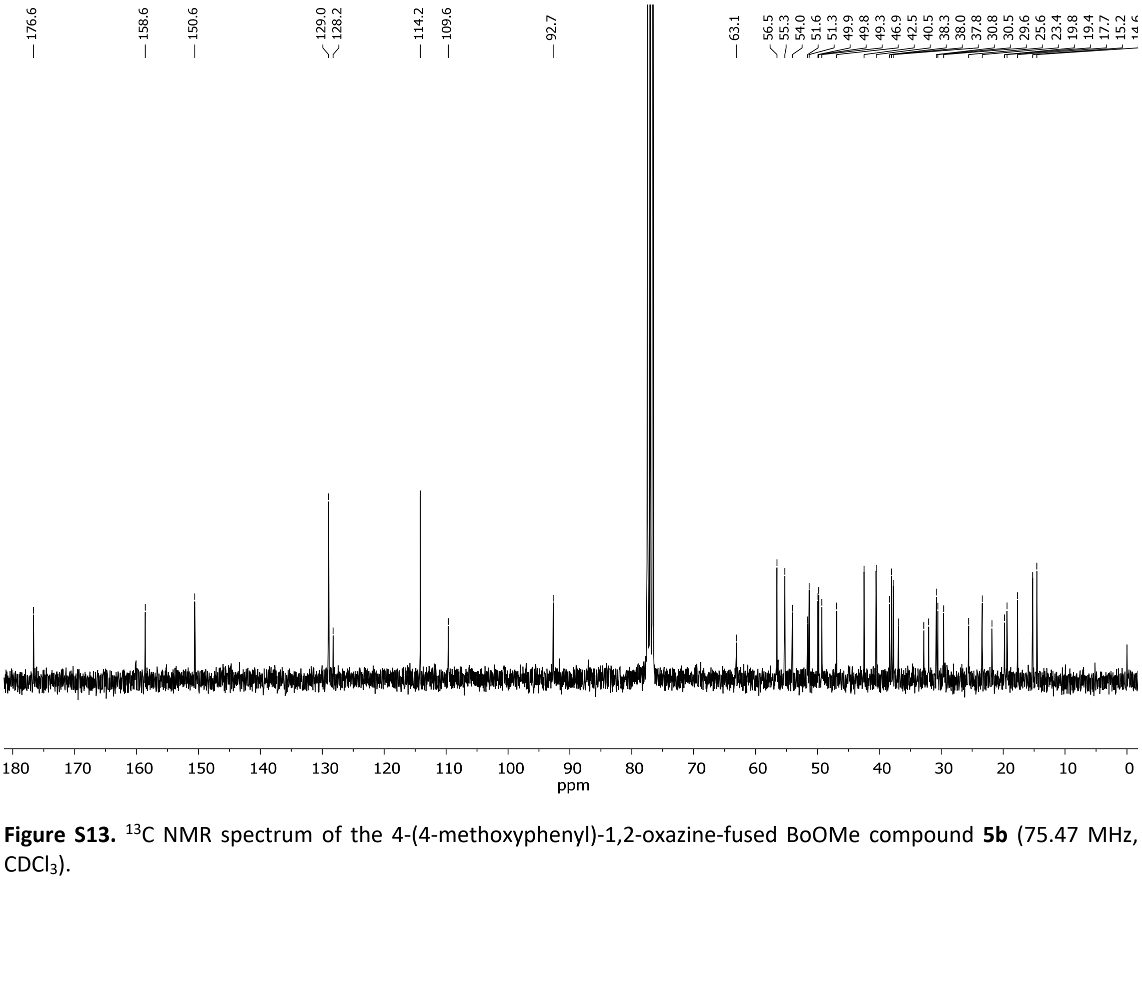

Figure S13. ^{13}C NMR spectrum of the 4-(4-methoxyphenyl)-1,2-oxazine-fused BoOMe compound **5b** (75.47 MHz, $CDCl_3$).

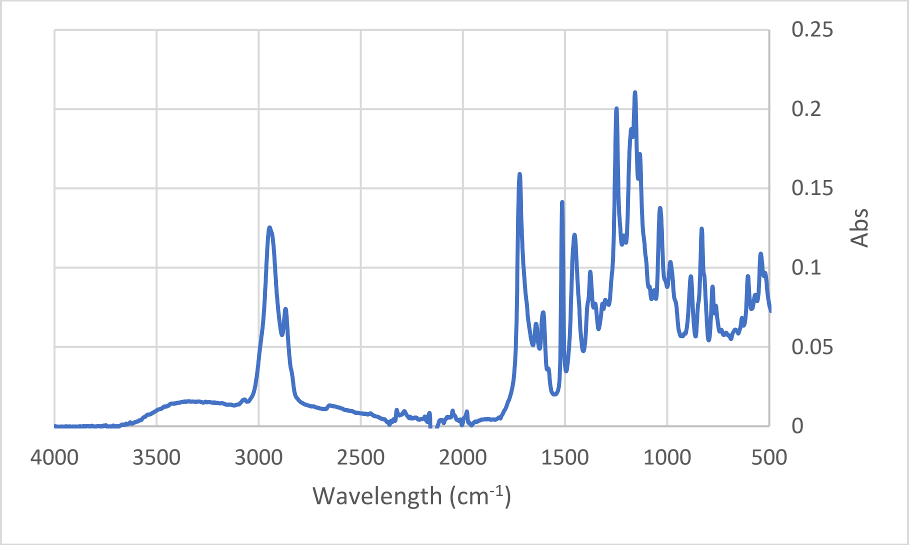

Figure S14. ATR-FTIR spectrum of the 4-(4-methoxyphenyl)-1,2-oxazine-fused BoOMe compound **5b** (FTIR Bruker Tensor 27, with ATR Golden Gate accessory, Diamond (Specac), Absorbance mode, resolution 4 cm^{-1}, 256 scans, 4000-500 cm^{-1}).

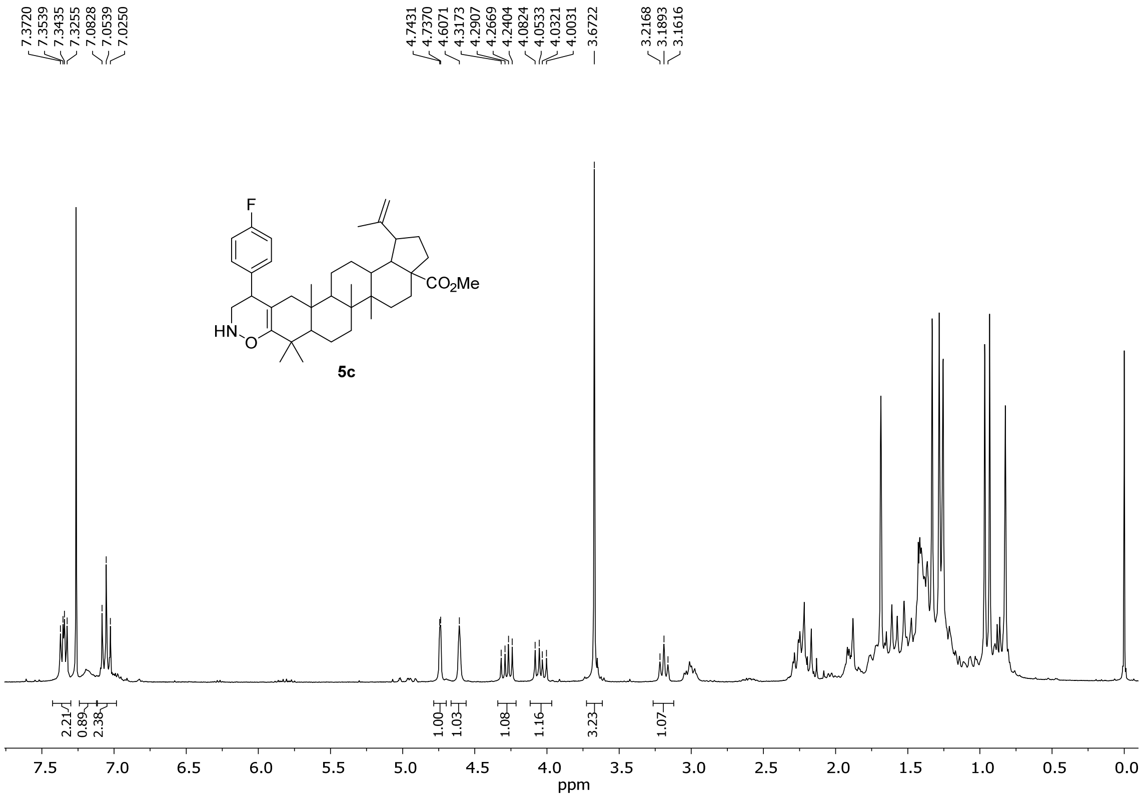

Figure S15. ^{1}H NMR spectrum of the 4-(4-fluorophenyl)-1,2-oxazine-fused BoOMe compound **5c** (300.13 MHz, $CDCl_3$).

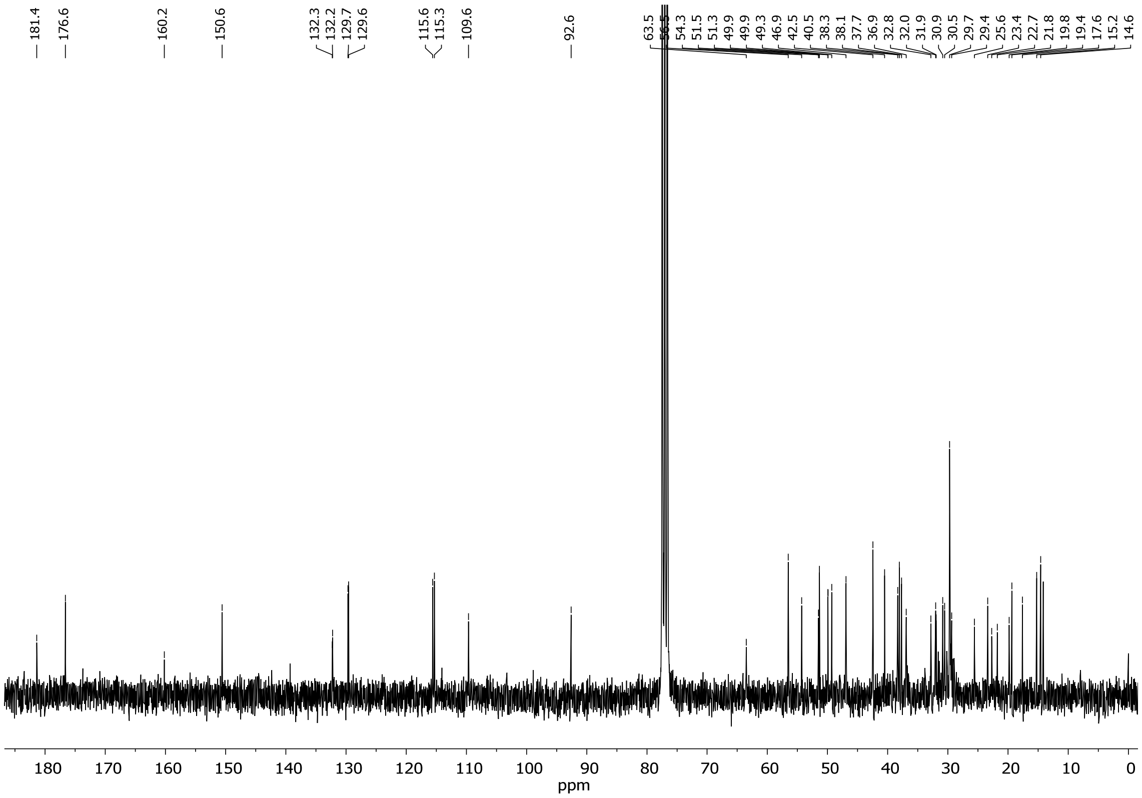

Figure S16. ^{13}C NMR spectrum of the 4-(4-fluorophenyl)-1,2-oxazine-fused BoOMe compound **5c** (75.47 MHz, $CDCl_3$).

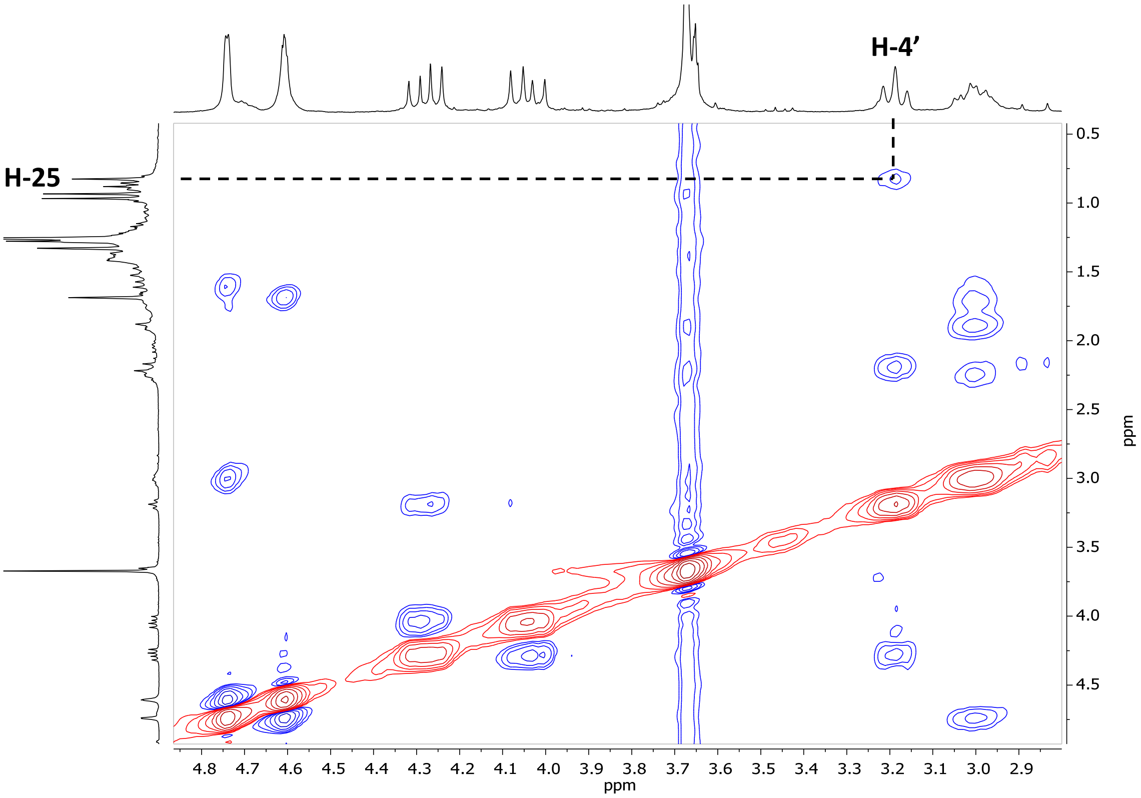

Figure S17. NOESY spectrum of the 4-(4-fluorophenyl)-1,2-oxazine-fused BoOMe compound **5c** (300.13 MHz, $CDCl_3$).

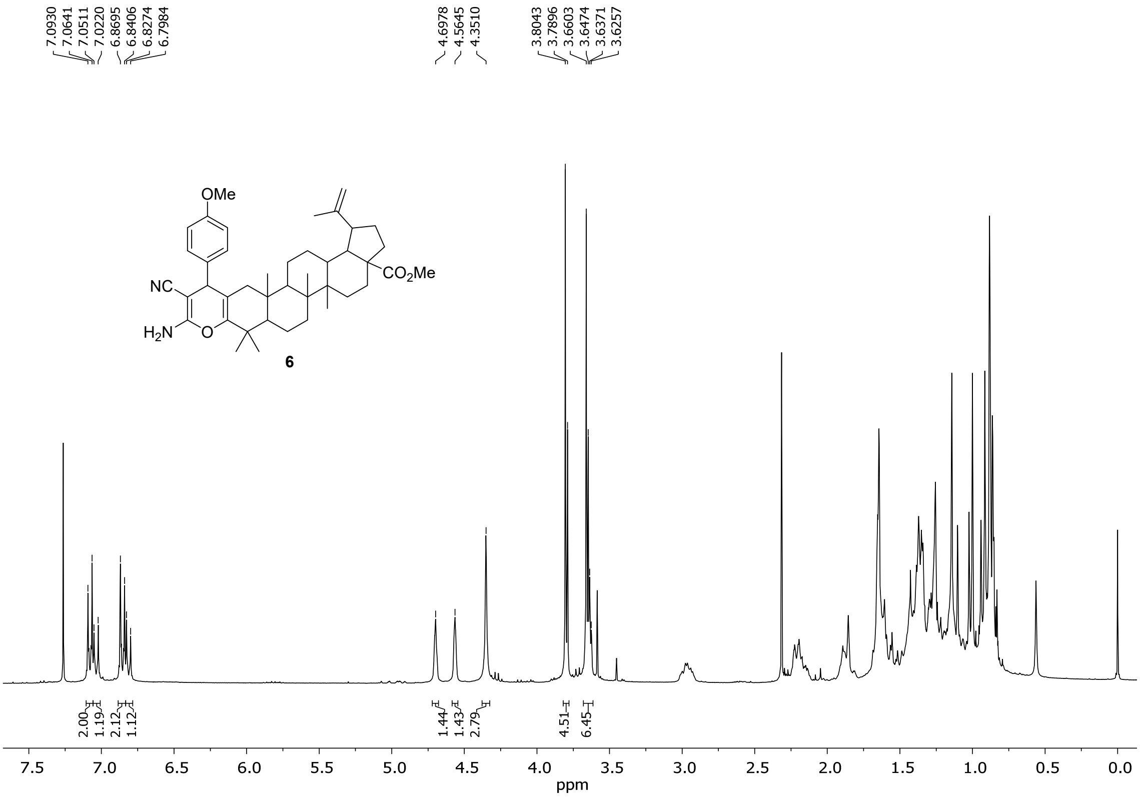

Figure S18. ^{1}H NMR spectrum of the pyran-fused BoOMe compound **6** (300.13 MHz, $CDCl_3$).

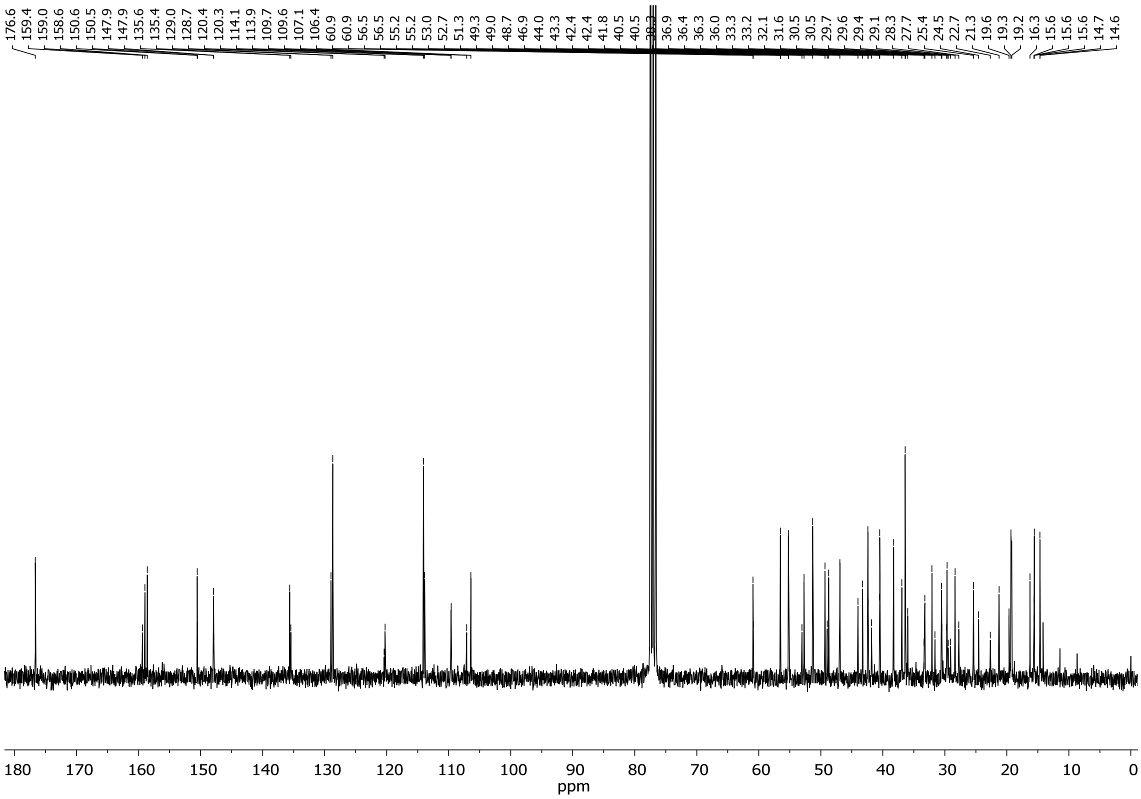

Figure S19. ^{13}C NMR spectrum of the pyran-fused BoOMe compound **6** (75.47 MHz, $CDCl_3$).

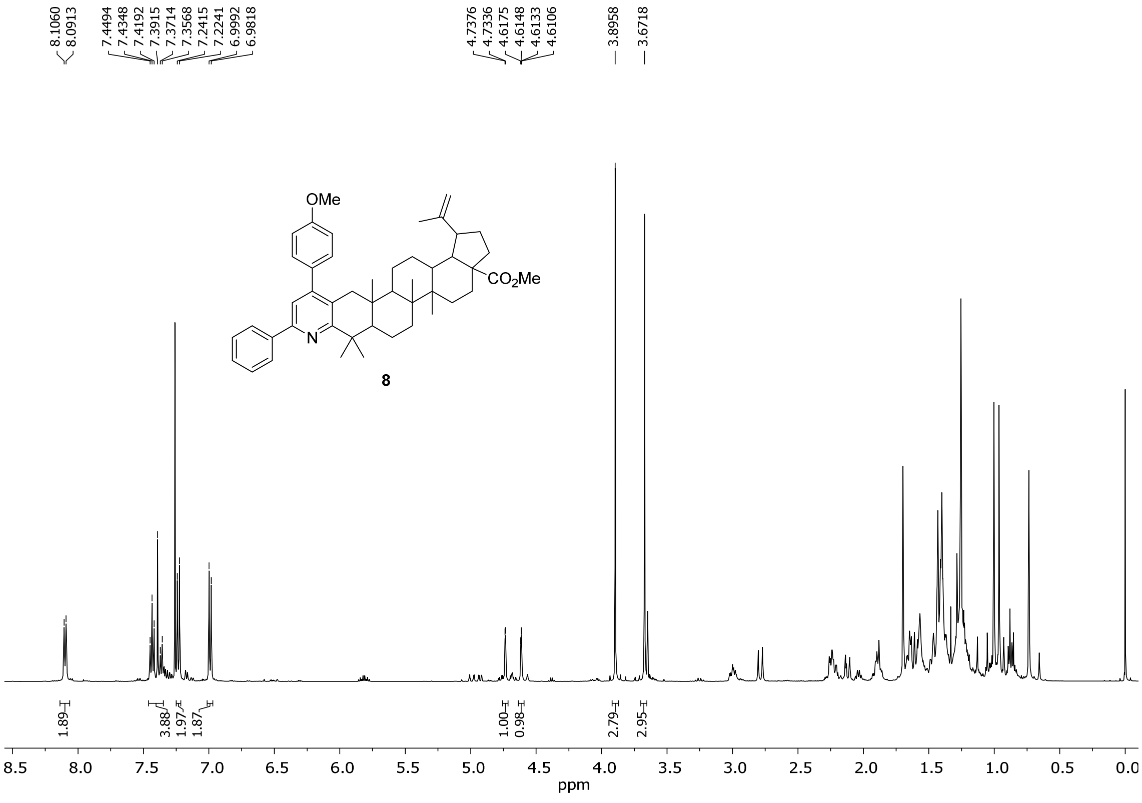

Figure S20. ^{1}H NMR spectrum of the diarylpyridine-fused BoOMe compound **8** (500.13 MHz, $CDCl_3$).

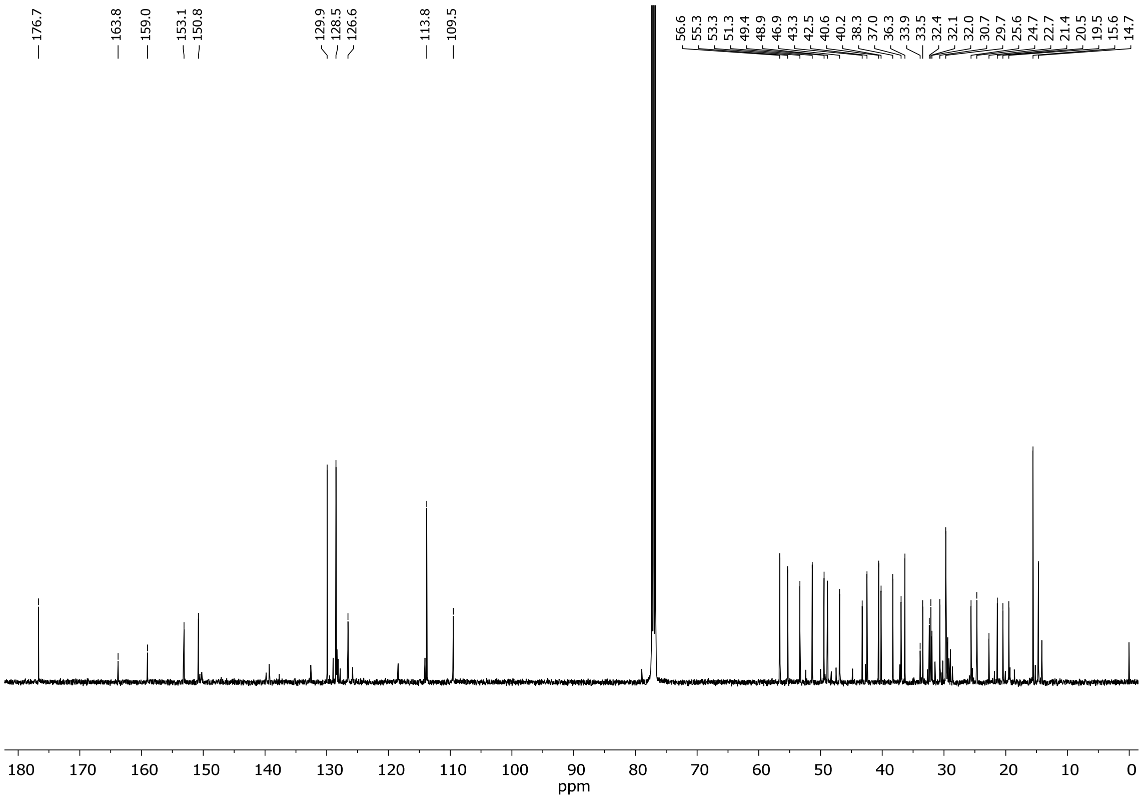

Figure S21. ^{13}C NMR spectrum of the diarylpyridine-fused BoOMe compound **8** (125.77 MHz, $CDCl_3$).

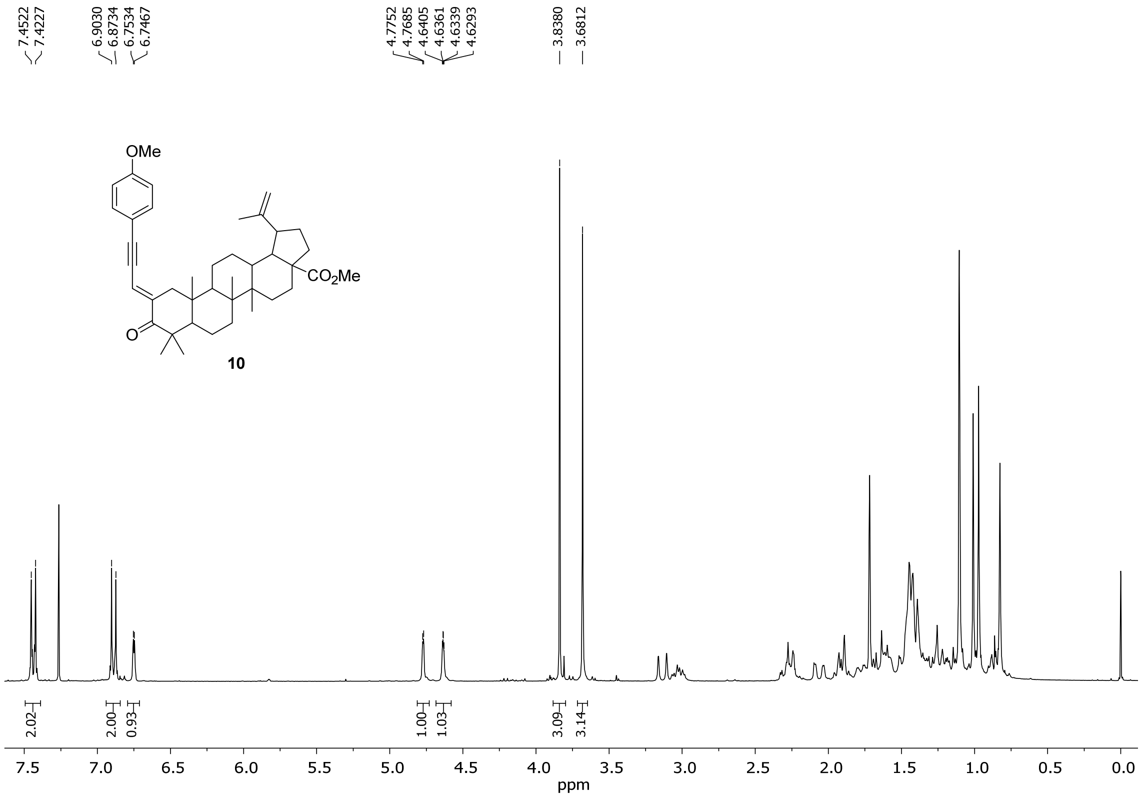

Figure S22. ^{1}H NMR spectrum of methyl (*E*)-2-[3-(4-methoxyphenyl)prop-2-yn-1-ylidene]betulonate (**10**) (300.13 MHz, $CDCl_3$).

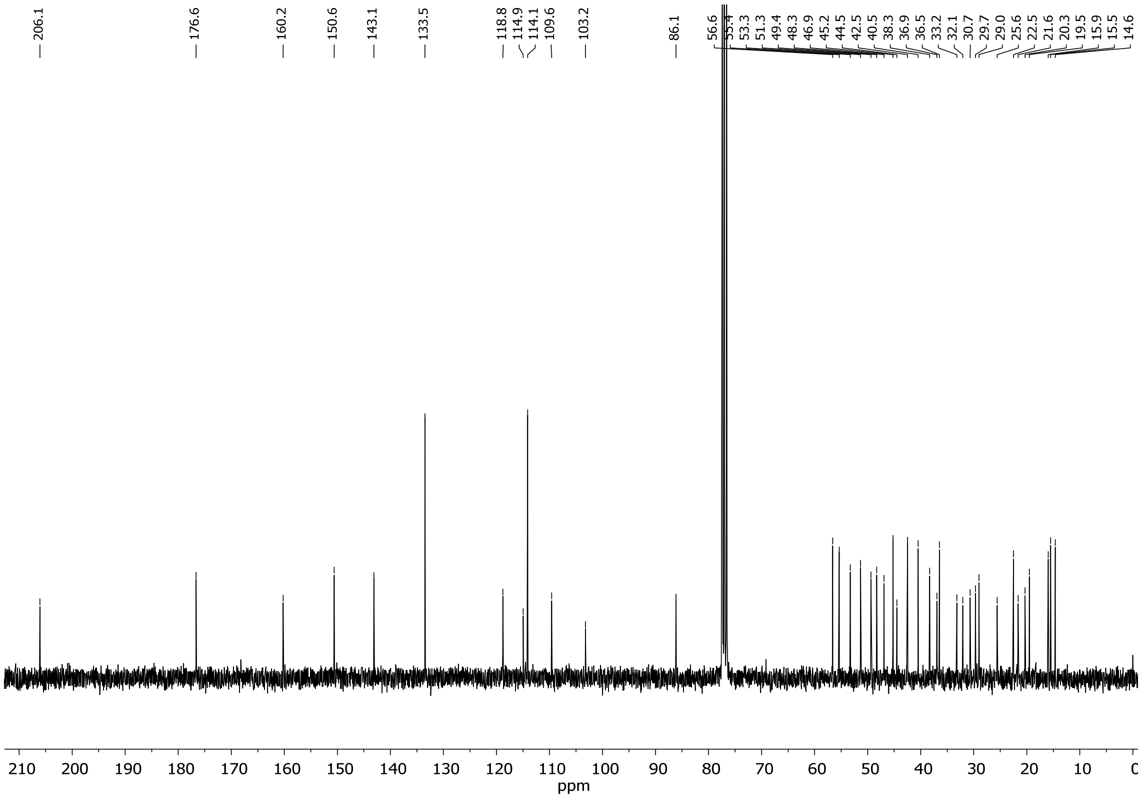

Figure S23. ^{13}C NMR spectrum of methyl (*E*)-2-[3-(4-methoxyphenyl)prop-2-yn-1-ylidene]betulonate (**10**) (75.47 MHz, $CDCl_3$).

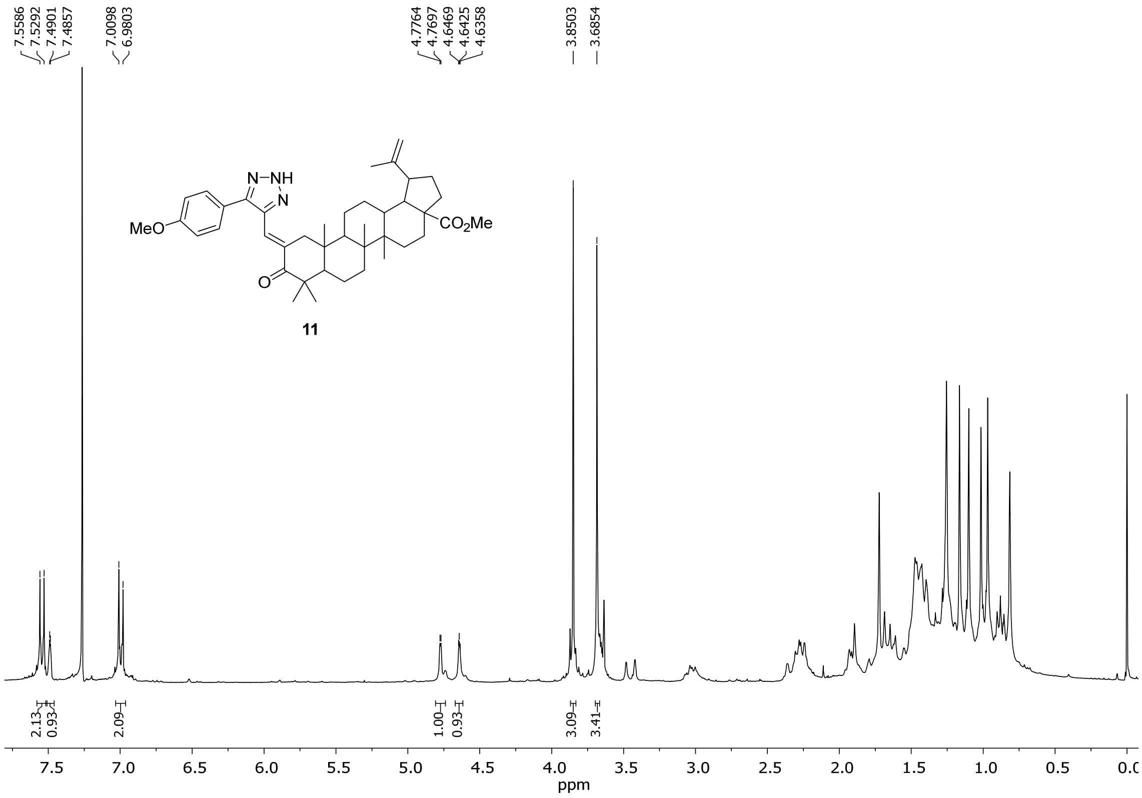

Figure S24. ^{1}H NMR spectrum of the 1,2,3-triazole–BoOMe compound **11** (300.13 MHz, $CDCl_3$).

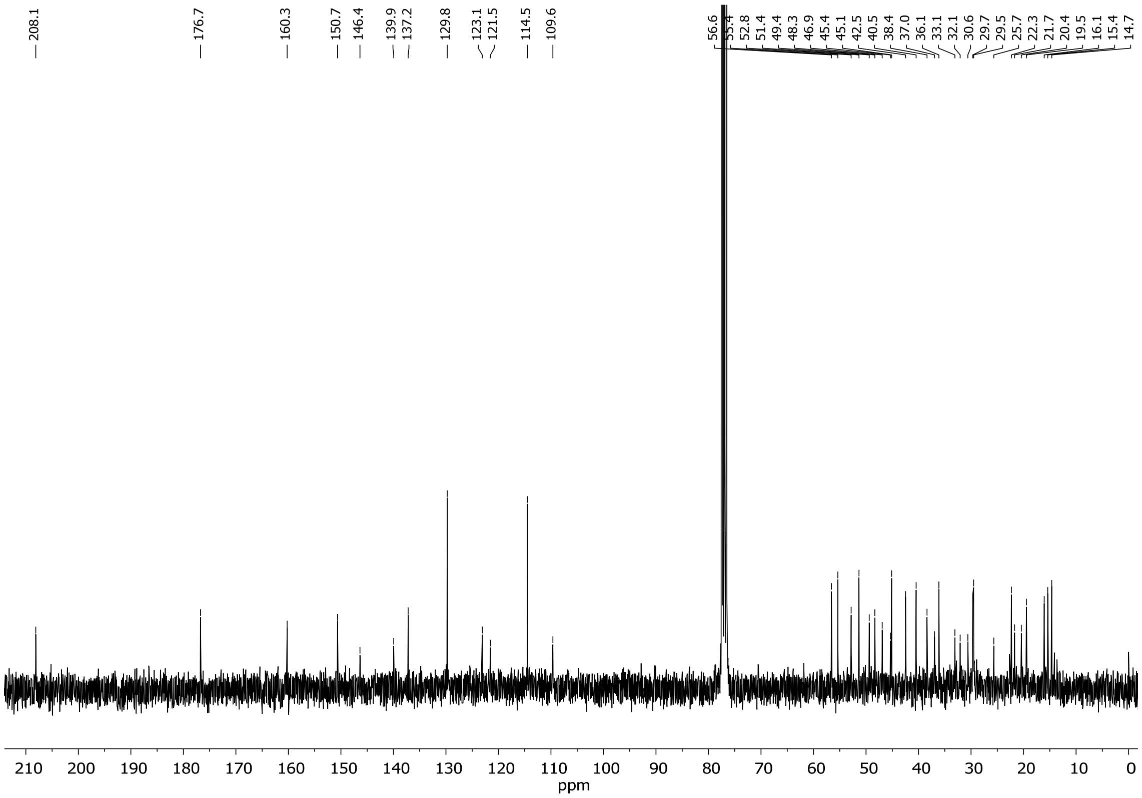

Figure S25. ^{13}C NMR spectrum of the 1,2,3-triazole–BoOMe compound **11** (75.47 MHz, $CDCl_3$).
